# Next-Generation Sequencing in Oncology: Genetic Diagnosis, Risk Prediction and Cancer Classification

**DOI:** 10.3390/ijms18020308

**Published:** 2017-01-31

**Authors:** Rick Kamps, Rita D. Brandão, Bianca J. van den Bosch, Aimee D. C. Paulussen, Sofia Xanthoulea, Marinus J. Blok, Andrea Romano

**Affiliations:** 1 Department of Clinical Genetics: GROW—School for Oncology and Developmental Biology, Maastricht University Medical Centre, 6229HX Maastricht, The Netherlands; rick.kamps@maastrichtuniversity.nl (R.K.); rita.brandao@maastrichtuniversity.nl (R.D.B.); bianca.vanden.bosch@mumc.nl (B.J.v.d.B.); aimee.paulussen@mumc.nl (A.D.C.P.); rien.blok@mumc.nl (M.J.B.); 2 Department of Gynaecology and Obstetrics: GROW—School for Oncology and Developmental Biology, Maastricht University Medical Centre, 6229HX Maastricht, The Netherlands; sofia.xanthoulea@maastrichtuniversity.nl

**Keywords:** next-generation sequencing, whole-exome-sequencing, whole-genome-sequencing, gene-panel, inherited cancer syndrome, cancer somatic mutation, diagnostics, genetic modifiers, theranostics

## Abstract

Next-generation sequencing (NGS) technology has expanded in the last decades with significant improvements in the reliability, sequencing chemistry, pipeline analyses, data interpretation and costs. Such advances make the use of NGS feasible in clinical practice today. This review describes the recent technological developments in NGS applied to the field of oncology. A number of clinical applications are reviewed, i.e., mutation detection in inherited cancer syndromes based on DNA-sequencing, detection of spliceogenic variants based on RNA-sequencing, DNA-sequencing to identify risk modifiers and application for pre-implantation genetic diagnosis, cancer somatic mutation analysis, pharmacogenetics and liquid biopsy. Conclusive remarks, clinical limitations, implications and ethical considerations that relate to the different applications are provided.

## 1. Introduction

Next-generation sequencing (NGS), also called massive parallel sequencing, was developed in the last decade and allows simultaneous sequencing of millions of DNA fragments without previous sequence knowledge. This advanced technology has been a true revolution compared with the traditional sequencing methods, in which one or a few relatively short fragments of DNA, previously amplified by Polymerase Chain Reaction (PCR), could be sequenced per tube. Due to the high costs and intensive work required, traditional sequencing was only performed on specific DNA regions and for specific samples. For instance, genetic screening of heterozygous mutations, such as in the case of breast/ovarian cancer or Lynch syndromes, was previously based on the screening of DNA heteroduplexes through different non-sequencing methods. Only selected samples from subjects with a strong indication for further DNA analysis would then be sequenced. Meanwhile, the Human Genome Project, which was launched in 1990, required 13 years and billions of euros in order to sequence the complete human genome.

With NGS, the today promise of today is that a complete genome can be sequenced in a few days for less than $1000 per genome. Even though we are not there yet, the implications and the impact of NGS in understanding the biological processes of diseases like cancer and in personalising patient care are unprecedented.

The present review describes the major milestones in NGS technology, the technical developments and application of NGS to the field of oncology, i.e., hereditary cancer syndromes and sporadic cancer, diagnostics, classification, therapeutics, theranostics and pharmacogenetics.

## 2. NGS—Next-Generation Sequencing Technology

The Sanger DNA sequencing method, also named chain terminator sequencing, was developed in 1997. This method, which was later automated and underwent slight modifications, was the sequencing gold standard until the late 2000’s [1]. Different approaches had meantime been developed and these technologies started to be implemented in commercially available NGS DNA sequencers. The first commercial NGS sequencer was based on pyrosequencing technology (developed in 1996) and was commercialized in 2004 Roche 454^®^ (Roche Diagnostics, Almere, The Netherlands). Since then, the costs associated with NGS have decreased continuously, with a massive decline during the last eight years, and several NGS sequencers with different chemistries have been launched into the market. Some advantages of NGS sequencers are the high-throughput sequencing capacity of large genomic regions or small regions for many samples and the fact that they do not require previous knowledge of the genome per se. Nowadays, the use of NGS almost replaced conventional Sanger sequencing and is a very versatile approach for several clinical and non-clinical applications.

### 2.1. NGS Technology, Historical Perspective and State-of-the-Art

A thorough overview of the different NGS platforms and approaches e.g., Illumina^®^ (Illumina, Eindhoven, The Netherlands), Oxford Nanopores^®^ (Oxford Nanopores, Oxford, UK), PacBio^®^ (PacBio, California, U.S.) or Roche^®^ (Roche Diagnostics, Almere, The Netherlands) is available at the “Next-Gen-Field-Guide” by Travis Glenn [2]. This synopsis was created in 2011, was last adapted in 2016, and specifies advantages, disadvantages, costs and overall performances of the different platforms.

Current NGS technology is sorted in two major types, i.e., short- and long-read sequencing. Short-read sequencing is mainly performed by Illumina^®^ protocols and machines and is described as cheap “sequencing by synthesis” (SBS) of reads shorter than 300 bp [3]. The ion semiconductor method (Ion Torrent^®^) is another cheap short-read sequencer [4]. The long-read sequencing is performed mainly by PacBio^®^ or Roche^®^, is a costly “single molecule real-time” (SMRT) technology of reads longer than 2.5 Kb [5,6]. The Oxford Nanopore Technologies^®^ MinION, using single stranded pore technology, actually allows to sequence very long molecules (>10 Kb) [7] and at a relative low cost but with a relatively higher error rate compared with other sequencers.

Short-read sequencing has low costs per Gb and high accuracy (low final error-rate; 0.1 Kb), and has been the method most frequently used until today. In contrast, the low accuracy (high final error-rate; >1 Kb) and the high costs per Gb of long-read sequencing make the use of these approaches non-versatile for all-purposes. Nevertheless, long-read sequencing presents unique advantages for a number of applications, because it improves the alignment in the bioinformatics pipeline (e.g., de novo assembly). Hence, it is more suitable than short-reads (even at low coverage) for familial haplotyping (allele phasing), to detect DNA and chromosomal structural variations, large chromosomal rearrangements, translocations and for the discovery of novel transcript-isoforms from RNA-seq data, allowing the detection of co-occurring splicing events. Recently, Qiagen GeneReader^®^ (Qiagen, Hilden, Germany) [8], 10x Genomics^®^ technology (10x Genomics, California, USA) [9] and Illumina NovaSeq^®^ sequencer (Illumina, Eindhoven, The Netherlands) [3] represent the latest developments in NGS. Qiagen GeneReader^®^ is a versatile system that provides the complete NGS pipeline from library preparation by PCR-based targeted exon enrichment to data analyses (see next paragraph) but can be coupled to other technologies and sequencers as well. The 10x Genomics^®^ technology coupled with Chromium System [9] allows making libraries suitable for multiple NGS platforms, linking reads into extra-long (synthetic) reads for analysis. The Illumina NovaSeq^®^ platform, launched in 2017, is a step closer to the promise of sequencing a human genome for less than $1000.

### 2.2. NGS Methods

**Figure 1:**
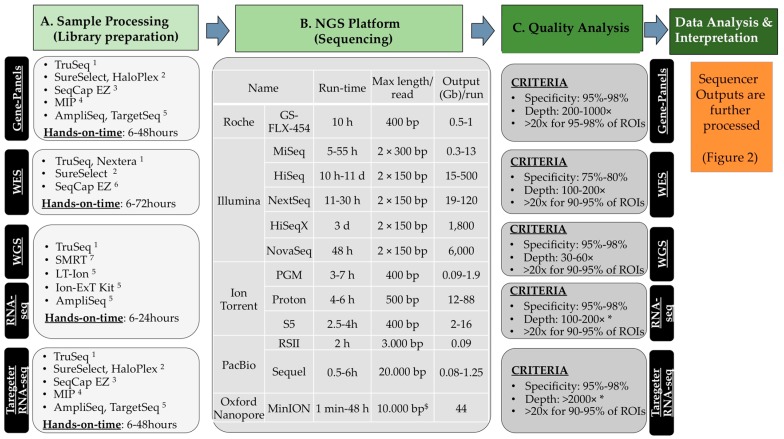
Pipeline illustrating the four major blocks in next-generation sequencing (NGS) studies. ^1^ Illumina^®^; ^2^ Agilent Technology^®^; ^3^ Nimblegen^®^; ^4^ MIP: Molecular Inversion Probe. This method is normally in house developed using specific tools (SciTools^®^, Integrated DNA Technologies, Coralville, Iowa, U.S.) assisting in probe design; ^5^ ThermoFisher^®^; ^6^ Roche^®^; ^7^ PacBio^®^. Because of their recent development, information about the Qiagen GeneReader^®^ and 10x Genomics^®^ technology are not included in this figure. ^$^ Users have reported up to 200,000 pb; * To detect low expressed transcripts, >2000× coverage is needed.

Different approaches can be used according to the needs and the questions to be addressed. The initial input material can be genomic DNA (DNA-seq), messenger or non-coding RNA (RNA-seq) or any nucleic/ribonucleic material obtained after specific procedures (see additional applications, Paragraph 6). The implementation of NGS technology can be visualised as four major blocks (Figure 1).

(a) Libray preparation or sample processing. The material is first fragmented mechanically or enzymatically to yield fragments whose size is compatible with the sequencer (small fragments of 200–300 nucleotides for short-read sequencing, longer for the long-read sequencing). This material can be enriched to analyse a limited number of genetic regions (e.g., disease gene-panels or microbes [10]) or all coding exons of the human genome (from approximately 21,000 genes; Whole-Exome-Sequencing, WES). The complete genomic DNA can also be sequenced (Whole-Genome-Sequencing, WGS) and it does not require any enrichment step (see Scetion 2.2.3). The regions that are intended to be analysed are defined regions of interest (ROIs). An amplification step through PCR with 4–12 cycles is perfomed in most cases. During this step, proper linkers and barcodes are attached to the DNA fragments and are necessary for subsequent analyses by the sequencer. DNA barcodes, which are unique nucleotide tags (6–8 nt), allow pooling samples together in one single flowcell for the sequencing reaction.
(b) Sequencing. Most common sequencers are described earlier [2,3,4,5,6,7] and Figure 1 summarises a few characteristics that distinguish them. A review on the different sequencing chemistries can be found elsewhere [11].
(c) Initial quality and raw data analyses. General quality control about the read quality is done mostly with FastQC [12]. Many pre-processing tools are available for removal of bad quality reads, trimming, etc. After mapping, specificity is determined, i.e., the fraction (%) of the total number of predefined ROIs, which are correctly enriched and sequenced.
(d) Variant calling and data interpretation. This last step is dependent on the specific application. In this review, some methods and bioinformatics tools relevant to data interpretation in the field of oncogenomics will be given.

#### 2.2.1. Gene-Panels

For most clinical applications, the use of gene-panels to sequence only a discrete number of genes of interest has been the method of choice, because of its cost-efficiency, and because at the same time it achieves high coverage of ROIs and offers simplicity in the raw and subsequent data analyses. When the number of genes sequenced is restricted to the few already analysed in previous diagnostic tests using traditional methods, this is normally called targeted re-sequencing (see also Section 3).

Different protocols are available to design and capture panels of genes and other ROIs. In most cases, companies providing the library preparation kits offer online user-friendly tools to design the hybridisation probes or the PCR oligos to enrich the desired ROIs. Enrichment can be obtained via solid phase hybridisation, in-solution hybridisation (most frequently used) or PCR-based enrichment and is followed by amplification via multiplex PCR, rolling-circle amplification (HaloPlex^®^) or amplicon-based-microdroplet-PCR (RainDance^®^ technology) [13]. The latter presents the advantage of simultaneously amplifying a large number of targeted regions into separate micro drops, thus keeping each amplification separate from the others and limiting the disturbance due to primer pair interactions. A cheap and flexible method to capture small regions of the genome for NGS analyses is the Molecular Inversion Probe (MIP) [14]. Table 1 describes the major characteristics of some of the gene-panels that have been developed in the field of hereditary cancer syndrome diagnostics through the last six years (the ROIs of these gene-panels are detailed in Table 2).

After sequencing, the analysis of raw data is relatively simple. Due to the high coverage per nucleotide, the specificity is high and only particular DNA regions may be captured inefficiently (due to high GC nucleotide content, for instance). Normally, sequencing depth of around 80× is sufficient to detect germline variants that are present in a homo- or heterozygous status. In case of somatic mutations, a higher coverage is required (>500) since mutations are usually present at sub-clonal levels resulting in low percentages.

#### 2.2.2. WES—Whole-Exome-Sequencing

Protocols/kits to enrich the library for all exons are available from several companies and use the same or similar technologies as mentioned for the enrichment of gene-panels. Following sequencing, raw data analysis is relevant in order to determine the quality of the experiments, checking for difficulties that may have occurred at the level of library preparation and/or sequencing. Both steps are crucial to obtain good quality data. A high sequence-on-target yield of more than 90% of the ROIs and coverage higher than 20× per nucleotide is necessary for sufficient specificity and sensitivity in mutation detection. Normally, when less than 90% of the ROIs are sequenced but coverage is high, sample processing was suboptimal; when the ROIs are sufficiently sequenced (>90%) but coverage is low, then the sequencing reaction was suboptimal and re-sequencing is required [15,16].

#### 2.2.3. WGS—Whole-Genome-Sequencing

In clinical diagnostics, in order to identify familial germline mutations, WGS may be useful in case genetic tests based on WES returned a negative result in families with a high probability of carrying a genetic mutation.

The major technical advantage of WGS is that the library preparation does not require any enrichment or amplification and the specificity is theoretically 100% (achieving around 95%–98% in practice, almost non-missing gaps) with a uniform coverage in the ROIs throughout the input material. Therefore, the possibility to miss a disease-causing variant (or any other information) for technical reasons such as inefficient probe targeted enrichment, inefficient amplification of a specific ROI or PCR amplification artefacts do not apply to WGS [16,17,18].

The most important obstacles in applying WGS on a routine scale are the high costs, the complex pipeline for data analyses and data interpretation. However, the more the costs of NGS decrease, bioinformatics tools improve and our understanding of the function of the non-coding part of the genome expands (also via the development of novel functional assays), the more this approach will be implemented. It is feasible that in the near future WGS will be performed routinely for every diagnostic question as a generic test and ROIs will be subsequently selected ad-hoc in silico for each specific application from the raw data, in order to find the causative genetic variants.

### 2.3. Data Analyses and Interpretation

**Figure 2:**
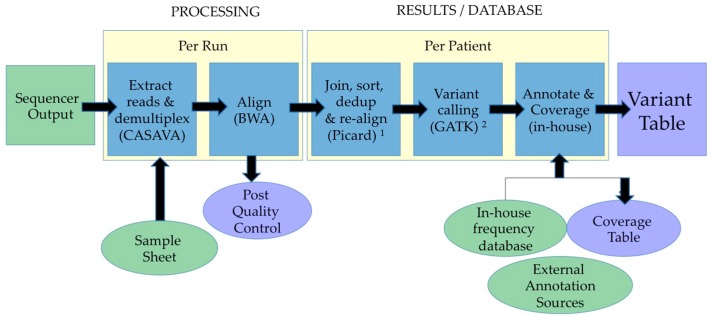
Data interpretation pipeline. Example of DNA-seq Bioinformatics Pipeline for Illumina^®^. ^1^ This steps removes duplicate sequences using the Picard program; ^2^ GATK: Genome Analysis ToolKit.

After raw data are assessed for sufficient quality, data analyses and interpretation continues using different pipelines depending on the approach used (gene-panel, WES, WGS or targeted-RNA-seq) and on the questions that need to be answered. Figure 2 illustrates briefly the major steps for DNA-seq used in genetic diagnostics to interpret data obtained from an Illumina^®^ platform.

Base-calling is performed using software like the Casava pipeline that produces Fast-Q files (raw-initial data), which can then be aligned to the human reference genome using Burrows-Wheeler-Alignment tool (BWA). Single base variants can be identified using Sequence-Alignment-MAP tools (SAM) and annotated. Additional software and scripts (normally in-house developed) match the data from NGS analysis to variants in reference databases (RefGene, dbSNP and UCSC genome browser [19]), in order to identify variants already annotated and identify new variants present in the NGS sample of diagnostic interest. This ultimately issues the “variant table” (Figure 1) that contains the list of all identified variants with, if available, information compiled from other databases such as allele frequency, pathogenicity, publications.

In case the experiment is aimed at identifying the genetic cause of an inherited disease in a patient or in a family, the goal is to assign a clinical significance to the identified variants. Several databases (OMIM [20] or LOVD [21]) catalogue known variants with respect to their pathogenicity.

Some additional annotated variants have not been characterised with respect to their clinical significance. For these and for new variants, several tools can be used that can predict their effect at the protein level, protein activity, RNA splicing and ultimately help assess the presence of protein damaging alterations (Alamut, Polyphen, Sift are some of these tools that are frequently used at clinical genetics laboratories [22]). Such analytical process can be performed manually for each variant or can be automated. Several guidelines are available to assist such classification (see Section 6). The ultimate proof of the pathogenicity of one variant and its risk to develop cancer (or any other genetic diseases) requires conducting functional analyses (in vitro, in vivo) and/or co-segregation studies of the variant with the disease among relatives. Similar considerations and sorts of analyses apply to studying and deciphering the effects of somatic mutations occurring in cancer.

### 2.4. Conclusions

Different methods and technologies are currently available to customise the use of NGS in order to fit each specific study. The costs of short-read sequencing are competitive with standard traditional methods. The next step in diagnostics (and other disciplines) will be to use WGS (eventually selecting ROIs in silico) and long-read sequencing (Oxford Nanopore^®^, PacBio^®^ or new inventor).

## 3. Inherited Cancer Syndromes

### 3.1. Historical Perspective in Inherited Cancer Syndromes

Several types of cancer display a familial predisposition and specific gene mutations confer a high-lifetime risk to develop the disease. During the last decades, the basis for such genetic predisposition has been clarified for several cancer syndromes and the high-penetrant/high-risk genes mutated in familial cases are currently subjected to genetic diagnostic screening programmes (Table 3). Mutation testing in these genes has major impact in genetic counselling, helps increase the chance of survival, defines the prognosis of carriers and identifies the most appropriate and personalised prophylactic measures [22,40,48]. In addition, in some countries, mutation-carriers can opt for in vitro fertilization (IFV) with pre-implantation genetic diagnosis (PGD) to prevent passage of the mutation to their offspring (see Section 3.6).

Hereditary breast and ovarian cancer (HBOC) and Lynch syndrome are among the most widely studied cancer syndromes. The high-penetrant *BRCA1* and *BRCA2* susceptibility genes for HBOC were discovered between 1994 and 1995. Subsequent genetic studies based on linkage and positional cloning helped identify additional moderate-risk genes [40,49,50] and genome-wide association studies identified common low-penetrance alleles associated with breast cancer heritability [51,52,53]. In Lynch syndrome, four mismatch repair (MMR) genes confer high-penetrance for colorectal and endometrial cancer onset (*MLH1*, *MSH2*, *MSH6*, *PMS2*) and intensive research explored and identified additional genetic risk variants [54,55,56]. In addition, several cancer syndromes confer high-risk for one cancer type but have also low penetrance and low- to moderate-risks for the development of tumours at additional sites (see Table 3) [24,57,58].

Although a number of moderate-risk variants has been identified and some have already clinical guidelines for genetic counselling, no additional high-risk casual genetic factor could be found besides the high-risk *BRCA1*/*2* and MMR genes in HBOC and Lynch syndromes [59,60]. The reason for not finding the genetic cause of hereditary syndromes consists in the fact that their genetic basis is more heterogeneous than initially thought, and besides the high- and moderate-risk mutations in known genes, variants in other unknown genes with moderate- to low-risk exist. Because of these reasons, genetic screening for hereditary syndromes based only on one or few genes is considered today no longer appropriate. NGS to explore the genome of families has started to unravel the genetic complexity and the basis of cancer syndromes.

This Chapter describes the impact of NGS on the care and management of heritable cancer syndromes, using cancer gene-panels (Section 3.2), WES/WGS (Section 3.3) and RNA-seq (Section 3.4). Focused cancer gene-panels have been most frequently used to date due to cost-efficiency, because the raw data handling and flow of data through bioinformatics pipelines is relatively simple, the demand for server data storage is limited and because they decrease the chance of finding variants of underdetermined/unknown significance (VUS), which are difficult to interpret in clinical management (see also Section 7). In this context, the most straightforward way to overcome these challenges is achieved by restricting the NGS sequencing to the high risk-genes only (targeted re-sequencing), which still presents some advantages and has higher sensitivity compared with traditional methods [61,62]. Although the use of WES and WGS has been less frequent in the past, the decreasing costs and the improvements in pipeline analyses are making these strategies increasingly more suitable and it is envisaged that they will be the preferred approach in the near future. Finally, novel approaches such as RNA-seq have the potential to identify genetic causes of cancer that are not recognised via screening of the genomic DNA.

### 3.2. Gene-Panels in Cancer Syndromes

Table 1 summarises some of the cancer-gene-panels that have been used preclinically and that are currently available for clinical use, with their major technical characteristics. Table 2 gives the list of genes captured by these panels (ROIs).

Before 2012, pre-clinical studies explored the performance, the validity and robustness of these approaches. In 2010, Hoppman-Chaney and colleagues [23] screened the DNA from five patients with Lynch syndrome who had already tested positive for a known MMR mutation. A solid-phase capture array was used to screen 22 genes, (including *MLH1*, *MSH2*, *MSH6*, and *APC*, Table 1 and Table 2) using two sequencing platforms (Roche 454^®^ and Illumina^®^). In this pioneering study, one indel mutation and a number of neutral variants previously identified with standard methods were missed by NGS. Depending on the platform used the sensitivity reported ranged between 90% and 93% and the positive predictive value was 78%–100%. These initial technical difficulties were related to suboptimal enrichment of GC-rich regions and to problems in the bioinformatics pipeline to correctly call indels, and were solved by subsequent improvements in capture protocols and data-analysis tools. In 2010, Walsh and colleagues from the University of Washington published a gene-panel for HBOC named BROCA, in honour of the French doctor Paul Broca who contributed to our knowledge of inherited breast and ovarian cancers. BROCA consists of 21 genes (Table 1 and Table 2) and was initially tested on 20 women with known inherited mutations. The protocol proved highly sensitive to detect all classes of mutations (single-base substitutions, small insertions and deletions, and large gene rearrangements) with zero false-positivity recovered [24]. Subsequently, the same panel was applied in a genetic screening of 360 women diagnosed with ovarian, peritoneal or fallopian tube carcinoma. Women were enrolled irrespective of family cancer history and 82 of them (23%) harboured one or more loss-of-function mutations (85 in total) that mapped in 12 genes. Most cases carried *BRCA1* gene mutations (11% of the subjects) followed by *BRCA2* (6%) and 10 additional genes (6%). Loss of heterozygousity in the wild-type allele was confirmed in more than 80% of the cases [25]. A similar approach was used by the same authors [27] to test a second seven-gene-panel named ColoSeq on 23 germline DNA samples from Lynch syndrome patients with a known germline mutation, 31 patient samples suspected of Lynch syndrome, 19 samples from subjects with no family cancer history, six samples from the HapMap project and three colorectal cancer cell lines known to carry MMR and/or *APC* mutations. NGS/gene-panel screen demonstrated 100% sensitivity to detect all sorts of alterations (nonsense, missense, frame-shift, in-frame deletions, splice site and large deletions and duplications) in the 23 patients with known alterations (all 23 mutations were detected) and in the five cell lines. Six novel pathogenic mutations and three VUS were identified in the prospective cohort of 31 subjects. The authors also subjected 75 samples twice to the NGS runs and demonstrated the technical reproducibility of the method, which increased as a function of the sequence reads (particularly for indels) and was 100% with reads over 40× depth.

These initial studies confirmed the concept that next to high-risk genes, like *BRCA1*/*2* and MMR genes, other genes also contribute to the familial cancer predisposition and undoubtedly demonstrated some potential of NGS to identify such genetic causes among families that test negative for mutations in high-risk genes using traditional methods. From March 2012 the use of gene-panels and NGS became clinically available and public and private centres started to use them for genetic testing. Panel-testing continues to improve in terms of genes screened, wet-lab technology, sequencers and bioinformatics pipelines (Table 1 and Table 2). The clinical implementation of gene-panels also means that large patient data-sets were generated and used to explore the specific penetrance of genes included in the panels and to assess the clinical utility, performance and implications of the use of NGS in diagnostics [64,65].

#### 3.2.1. Technical Validity

The technical validity of gene-panel/NGS in comparison with the reference technology was thoroughly assessed in different studies [29,36,42,66,67,68,69,70]. In particular, one of these assessed the sensitivity and specificity of NGS compared with standard Sanger sequencing and MLPA (multiplex ligation-probe amplification) and also confirmed the performance of three different panel designs for HBOC (Custom Design by Castera et al. [29]: Table 1 and Table 2). A 100% concordance of gene-panel/NGS to detect SNVs, indels or large rearrangements was demonstrated on an initial series of 59 patient DNAs harbouring known germline mutations of *BRCA1* or *BRCA2* genes and it was confirmed on 168 additional samples [29]. Complete concordance with the reference methods was also demonstrated using a 29-gene-panel on 1062 subjects, in which over 750 variants were identified, including technically challenging classes of sequence and copy number alterations [42]. The reproducibility between two separate German centres that used different gene-panels and library preparation methods was tested on 12 specimens with over 99.5% concordance (one non-pathogenic variant was called in discordance between the centres and it turned out that this specific alteration mapped in a region without sufficient coverage) [39].

In genetic diagnostics, the detection of gene variants in *PMS2* and *CHEK2* is challenging due to the presence of pseudogenes. Although for routine diagnostics many centres use traditional techniques [31], NGS also demonstrated accuracy in detecting these alterations through the incorporation of long-range PCR that yielded a full (100%) concordance with Sanger sequencing, MLPA and comparative genomic hybridisation (CGH) [36,37,38].

#### 3.2.2. Clinical Relevance

One of the first and largest surveys conducted, reviewed the results of the first 2079 patients who received gene-panel testing (14 to 21 gene-panels) at Ambry Genetics [32]. All subjects were referred for genetic testing by their physician because of hereditary cancer. Distinct gene-panels, depending on the family history/cancer site, were used and all panels identified a high proportion of patients carrying a pathogenic/likely pathogenic variant (7.4% for BreastNext, 7.2% for OvaNext, 9.2% for ColoNext, and 9.6% for CancerNext; Table 1 and Table 2) [31].

Numerous additional publications confirmed this potential utility. In HBOC, all studies consistently indicated that genes besides *BRCA1* and *BRCA2* are mutated and confer a moderate- to high-cancer-risk. In a study on 708 consecutive patients suspected of HBOC, besides 69 germline deleterious alterations in *BRCA1* and *BRCA2*, additional putative pathogenic mutations were identified in *PALB2* (almost 1% of the patients), *TP53*, *CHEK2*, *ATM*, *RAD51C*, *MSH2*, *PMS2* and *MRE11A* (between 0.4% and 0.7% of the patients), followed by *RAD50*, *NBS1*, *CDH1*, *BARD1* (about 0.1%) [29]. Tung and co-workers [38] tested a 25-gene-panel on 1781 breast cancer patients who were referred for *BRCA1*/*2* gene testing at Myriad Genetics [35] and found that 9.3% of the patients harboured a mutation in the *BRCA1*/*2* genes, but 4.2% of the patients carried a mutation in 14 additional genes. The most frequent mutations (3.9% of the patients) were found in *PALB2*, *CHEK2* and *ATM*. In another group of 377 patients with a personal history of breast and/or ovarian cancer who were negative for *BRCA1/2*-mutations (BRCAx), 5% of the subjects carried a mutation in genes other than *BRCA1/2* [38]. Also Ambry Genetics, using a 19-gene-panel (OvaNext; Table 1 and Table 2) on 911 BRCAx subjects detected a mutation in over 7% of the subjects. *CHEK2* (2.5% of the patients), *ATM* (0.8%), and *TP53* (0.8%) were the most frequently mutated genes in breast cancer, whereas ovarian cancer patients carried mutations in *BRIP1* (1.7%) and *MSH6* (1.3%) [34]. Similar results were obtained by other centres [39,61] including Invitae [41], where 1062 subjects tested with a 29-gene-panel because of HBOC confirmed *ATM*, *PALB2*, *CHEK2* and some MMR genes as the most frequently mutated genes, plus *MUTYH* [42] with a possible recessive behaviour. Also, when a higher number of genes was screened, mutations were detected in the same set of genes. Aloraifi and co-workers [40] sequenced 312 genes among 104 BRCAx subjects and found that 13 subjects carried a pathogenic variant, most frequently occurring in *ATM*, *RAD50*, *PALB2*, *CHEK2* and *TP53*. Target-enrichment of candidate genes allows increasing the number of families for whom a causative variant is found with an extremely fast approach. This is particularly important because the individuals from these families will benefit from adequate medical advice.

Similar conclusions apply to syndromes associated with colorectal, endometrial and other gynaecologic cancer risk. Panel testing is suitable to identify pathogenic mutations in over 10% of the subjects with colorectal cancer, as indicated in a series of 586 patients with a personal history of the disease and indicated by their physicians for gene-panel testing at Ambry Genetics [33]. Additionally, mutations in genes other than the high-risk genes can be identified: in a cross sectional study, 1615 subjects with a history of Lynch syndrome associated cancer (patients had been previously referred by their physician for MMR and *EPCAM* aberration testing at Myriad Genetic) were further anonymously screened using a 25-gene-panel. In a group of 1112 patients with criteria for Lynch syndrome testing, 114 subjects had mutations in one of the Lynch syndrome genes and 71 carried mutations in other cancer predisposition genes, such as breast/ovarian cancer genes. Retrospective analysis identified that a small group of these patients also met the *BRCA1*/2 screening criteria. For every five Lynch syndrome mutations, a mutation was found in another gene [37]. This would have not been possible without the use of a gene-panel. Similarly, Fray and co-workers [84] focused on 127 patients with hereditary gynaecological cancers who underwent gene-panel testing because singe-gene test was negative, and 7% of the patients revealed the presence of a pathogenic mutation outside the expected high-penetrant genes.

#### 3.2.3. Cancer Gene-Panels: In Conclusion

Gene-panel testing in genetic diagnostics offers a cheap, reliable, fast and easy approach for hereditable cancer syndromes. The diagnostic yield is increased compared with singe/few gene testing. The most comprehensive analysis on the performance of gene-panels has been recently published and describes the results on 10.030 consecutive patients referred to GeneDX [44] because of inherited breast, ovarian, pancreatic, colorectal and endometrial cancers [45]. A molecular diagnosis could be made in 9.0% of the patients on average. The positive yield was 9.7% for breast, 13.4% for ovarian and 14.8% for colon/stomach cancer, and was highest for Lynch syndrome/colorectal cancer. Gene-panels identify a pathogenic or likely pathogenic mutation in over 8%–15% of the cases, therefore, gene-panels provide a broader picture on the genetic heterogeneity of cancer syndromes and identify mutations in genes that might not be tested otherwise. Although several putative pathogenic mutations in new genes have been characterised, the pathogenicity in most cases was not further confirmed by segregation analyses [29]. This becomes relevant especially because, when compared with single gene test, or with re-sequencing of high-risk genes only, gene-panels/NGS return a large number of VUS [31,33,37,42,84], which pose relevant clinical issues (see Section 7) and whose pathogenicity need to be confirmed. This review focuses on oncogenomics, nevertheless gene-panels and NGS is appropriate for non-oncological conditions as well. Companies providing genetic-test services offer gene-panels for over 100 conditions and custom design, including ways to personalise the capture design.

### 3.3. WES and WGS

Depending on the disease, between 70%–92% of the patients remains mutation-negative or undiagnosed after gene-panel testing [31,45]. It is possible that mutations in genes (or regions) not included in the panels contribute to the cancer risk and complete exome- or genome-analyses through WES or WGS are more appropriate tools to explore the genetic basis of familial syndromes.

WES and WGS studies identified new high- and moderate-risk genes in several types of cancers, such as the pancreatic cancer susceptibility genes *PALB2* and *ATM* [85], the hereditary pheochromocytoma susceptibility gene *MAX* [86] or the hereditary colorectal cancer moderate-risk genes *POLD1* and *POLE* [87]. Several studies identified novel HBOC susceptibility genes like *XRCC2*, *FANCC* and *BLM* [88,89,90,91,92,93]. Although it is feasible to apply WES and WGS in the near future as a generic test for every genetic diagnostic question, due to the costs (still high), the need of complex bioinformatics pipelines, of large storage capacity and the expected high number of VUS detected, today the clinical utility of mutation discovery throughout the complete exome or genome analysis is not convenient yet [91,94] and should be directed to specific patient groups [95].

### 3.4. Identification of Pathogenic Germline RNA-Splice Mutations Using RNA-Seq

Splicing, the process of removal of the introns in the pre-mRNA molecule leaving the exons adjacent to each other in the mRNA molecule is highly regulated by the RNA-splicing machinery and depends on specific genetic sequences to mark intron/exon junctions. Additionally, epigenetic factors (chromatin conformation and histone modifications) have also been implicated in the regulation of splicing [96]. Almost all human genes have different isoforms due to alternative splicing. Alternative splicing increases the number of proteins that can be produced from one single pre-mRNA molecule, which contributes to biodiversity, as these may have diverse, even opposite, functions. However, incorrect splicing, which occurs due to the presence of genetic variants, has been implicated as the hereditary cause of many genetic diseases, including hereditary cancer syndromes. We and others have detected *BRCA1*/*2* germline variants that lead to aberrant splicing and are, therefore, pathogenic [97,98,99,100,101,102]. Germline mutations that affect RNA-splicing are found in many other cancer genes, like *RAD51B* and *CDKN2A*, which play a role in familiar melanoma and pancreatic cancer cases [103,104,105,106]. Splicing mutations are also frequently found in *NF-1*, causing neurofibromatosis type-1 [107].

The genetic screenings used in the clinical setting are mainly DNA-based sequencing approaches and are usually limited to the protein coding region (exons) of the genome, missing, for example, introns, promoter regions and non-coding RNAs, which are important for gene-expression regulation. In addition, computer-based tools and pipeline analyses do not predict accurately the effects of the majority of the variants on gene-expression or RNA splicing. Therefore, mutations with a potentially pathogenic effect on RNA expression or processing are either not included in the regions of interest during sequencing or are filtered out during bioinformatics analysis since the effect on RNA cannot be properly assessed in silico. Putative splice variants might be common among the high number of VUS that are identified with NGS [31,33,37,42,84].

Ad-hoc experimental confirmation for each variant is laborious and time/consuming, and therefore not practical. Sequencing the transcriptome through RNA-seq can avoid these experiments and return an immediate result about abundance of a pathogenic RNA species or the formation of pathogenic splice variants. RNA-seq allows the analysis of the transcriptome at an unprecedented coverage [108,109,110,111] and can provide new insights into disease onset. Similar to WGS and unlike Sanger sequencing, it does not depend on previously known sequences, which is of particular relevance when studying the mRNA and its alternative splicing events. RNA contains information about nonsense, missense, silent, in-frame and frame-shift mutations, as can be observed at DNA-level, as well as splicing and allelic gene-expression changes, which are missed by DNA analysis. The use of RNA-seq has led already to the identification of new non-coding RNAs, gene fusions (in tumours), gene isoforms—through additional/changed promoters, exons or 3′ untranscribed regions—as well as the quantification of alternative RNA-splicing events by quantifying splice-junctions. Furthermore, mutations in transcription factor binding sites and promoter regions, and aberrant methylation can also be deduced, through thorough analysis of RNA-seq data. In most cases, it is not possible to know which splicing events occur together giving rise to the same transcript. But this limitation can be overcome by the use of single-molecule long-read sequencing, used by Pacific Biosciences^®^ and/or Oxford Nanopore Technologies^®^ instruments, or through synthetic long-read sequencing using a modified Illumina^®^ protocol or 10x Genomics^®^ chemistry (described in Section 2).

One challenge of RNA-seq analysis involves RNA-editing. This is a rare process in vertebrates that mostly causes substitutions in repetitive elements. In humans, RNA-editing occurs in less than 0.01% of the nucleotides and only about 1% of these events occur in non-repetitive regions [112]. Yet, it could lead to misinterpretation of the data. Therefore, although rare, it is important to take RNA-editing into account. Current available options to identify variant changes due to RNA-editing include consulting the Database of RNA editing in flies, mice and humans [113] or using a genome-independent tool to assess RNA editing sites [114]. Combining RNA-seq with WGS protocols and integrated analyses will also aid identification of RNA-editing events and will increase the chance of finding the causative DNA variant.

Despite the great advances in RNA-seq, this technology is not yet commonly used in routine clinical genetic screening. This may be for a number of reasons. The fact that RNA is less stable than DNA and requires higher care in handling and storage of the samples may be one of the reasons. In addition, RNA expression (and processing) is tissue-dependent and the tissue that one aims to study may not contain the RNA of interest. Also, data-analysis tools and trained personnel to use these tools and interpret the data are not always available. However, for genes expressed in tissues that can be collected with minimal invasiveness such as blood (notably, DNA repair genes involved in cancer syndromes are expressed in leukocytes), it can be advantageous (cost- and time-wise) to sequence the transcriptome. As a matter of fact, several studies have shown the benefit of RNA-seq in screening splice variants in inherited diseases. Recently, a publication reported the success of transcriptome sequencing to increase mutation-detection rate in undiagnosed rare neuromuscular disorders [115]. Also, it is noteworthy that RNA-seq studies in Huntington’s disease (an autosomal dominant genetic illness that causes degeneration of the brain nerve cells) have compared the splice isoforms in the brain of patients with those in controls and identified altered expression of splicing factors [116]. These splicing factors are now putative novel treatment targets.

Similarly, the identification of specific splice isoforms or altered splice factors in cancer syndromes are expected to lead to a better understanding of the pathologies and to new treatment opportunities. Several treatment options already exist to overcome genetic defects that affect splicing. For example, treatment with antisense oligonucleotides has already shown promising results [117,118] and the SMaRT technology (spliceosome-mediated RNA trans-splicing) has also been investigated for the treatment of at least ten diseases [119,120].

In brief, genetic diagnostics would benefit from RNA-seq approaches to allow the detection of a higher number of mutations involved in disease onset than currently possible and this will aid in improving personalised care and medical management.

### 3.5. Risk Modifiers

The identification of a germline mutation in known low-, moderate- or high-risk genes is important to explain the cause of hereditary cancer syndromes and has a clear positive benefit for patient care. However, during patient counselling, the incomplete and variable penetrance of pathogenic mutations poses problems on patient risk-management. For instance, a mutation in *BRCA1*/*2* confers a variable lifetime-risk that can be over 80% (ranging from about 40%) for breast cancer [24] and 50%–60% for ovarian cancer (in case of *BRCA1* mutation, lower risk for *BRCA2* mutation carriers; Table 3 and [25]). Similarly, a subject carrying a Lynch Syndrome mutation in one MMR gene has about 50%–80% lifetime-risk to develop colorectal cancer (in case of a mutation in *MLH1* or *MSH2*, but lower risk is associated with *MSH6* and *PMS2* mutation carriers), and 15%–70% risk of endometrial cancer in women (Table 3 and [121]). In addition, several cancer syndromes confer a high-risk for some tumours but also a moderate- to low-risk for tumours at other sites (Table 3). For example, a low-risk for breast cancer is present among carriers of mutations associated with Li-Fraumeni, Cowden, Peutz-Jeughers, and Hereditary Diffuse Gastric Cancer syndromes (Table 3 and [24]).

It is accepted today that several factors can modify the penetrance of a pathogenic mutation. Environmental factors play an important role but also genetic risk-modifiers exist [55,64,122,123], and their analyses and identification can help assess patient risk and aid counselling activities.

Genetic risk-modifiers are variants with no intrinsic pathogenic action, but they can modify the penetrance of a pathogenic mutation. Therefore, they have no effect on the general population, but only in subjects who already carry a cancer-risk aberration. Genetic risk-modifiers do not map necessarily on cancer-related genes, but also on other genes controlling various physiological pathways and cellular signalling. The study of genetic risk-modifiers is complicated because of their small effect-size. In addition, most studies examine the cancer onset as an endpoint, which is primarily caused by the pathogenic mutations in high- and moderate-risk genes. Only few studies assessed other clinical parameters, which can be influenced by a co-existing risk-modifier.

In most cases, the search for risk-modifiers has focussed on common SNPs through the use of SNP-arrays and genome-wide-association-studies (GWAS) [54,122], however, a recent study demonstrated that NGS is also a suitable approach to search for genetic risk-modifiers [55]. A family-based study was used to screen 154 genes among 35 endometrial cancer patients carrying a Lynch syndrome mutation. A number of genetic variants were identified and were separated in those with no effect and those with putative risk-modifying action. The occurrence of an increasing number of risk modifying-variants was associated with poorer clinical characteristics in patients (i.e., early age at diagnosis and the diagnosis with multiple cancers [55]). This was in line with other investigations based on SNP analyses [54].

Aloraifi and co-workers [40] applied to HBOC subjects a gene-panel that included cancer-associated but also a large number of non-cancer related genes. They used a 320-gene panel including the classical high-risk genes (*n* = 8), genes identified as cancer-related by GWAS (*n* = 88), those somatically mutated (*n* = 33), or methylated (*n* = 48) and 125 genes involved in cellular signalling. They found that 16% of the subjects carried a potential damaging alteration in genes not involved in cancer. However, the aim of the study was to identify additional HBOC susceptibility genes and the co-occurrence of various genetic aberrations including putative risk-modifying variants with respect to the patient clinical features was not explored. Several additional studies identified variants that could have risk-modifying effect but the authors do not further explore the co-occurrence of these variants with a pathogenic mutation and the association with patient clinical features [31,42,84]. In a recent large analysis of gene-panel testing for cancer syndromes [45], although the authors did not search specifically for risk-modifiers, it was found that over 3% of the subjects were carriers of a high-risk (pathogenic) mutation in combination with one or two additional aberrations in genes that were classified as moderate- or unknown-risk. Over 20% of these multiple mutation carriers reported multiple primary tumours.

### 3.6. Prevention of Inherited Cancer Syndromes

Once a definite genetic diagnosis for an inherited cancer syndrome has been made, future parents may wish to avoid transmission of the predisposition to their offspring. As such, couples have different reproductive options including (1) refraining from having children; (2) child adoption; (3) use of oocyte or sperm donor; (4) prenatal diagnosis (PND) and (5) preimplantation genetic diagnosis (PGD) in combination with an in vitro fertilisation (IVF) procedure. If there is a wish for a biologically “own” child, choices are between diagnosis either before or after conception. PGD is nowadays considered a well-established clinical service in many countries, where it is indicated for many inherited cancer syndromes such as familial adenomatous polyposis coli (FAP) [124], neurofibromatosis [125], Von Hippel-Lindau syndrome, retinoblastoma, Li-Fraumeni syndrome [126], tuberous sclerosis and HBOC [127].

The PGD procedure for monogenic diseases involves IVF via intra cytoplasmic sperm injection (ICSI), followed by genetic analysis of polar bodies or one or two blastomeres biopsied from a 4–10 cell cleavage stage embryo on day 3 post fertilization [128]. Depending on the PGD/IVF centre procedures, transfer of an unaffected embryo is performed on either day 4 post fertilisation or at a later stage when embryos are frozen after biopsy. In the early days, the genetic test included a PCR-based mutation specific test, soon including one or two genetically linked markers to control for allelic drop-out, a phenomenon specific to single cell analysis, and recombination events [129]. As the development of these mutation-specific tests required a continuous novel design and optimisation at the single cell level and is therefore both labour-intensive as well as expensive, PGD centres gradually tried to make the tests more “universal” by using multiplex microsatellite marker amplification determining the indirect risk haplotype, also called preimplantation genetic haplotyping (PGH; [130,131]). This method could be used for many families with the same genetic disease, without the former costs and time spent on each individual couple/family. There are however some limitations to this technique including in some set-ups the necessity of a whole genome amplification and the fact that the test can only be used in familial diseases, where the risk haplotype is deduced from available family members.

This PGH method was a huge improvement in many ways (robustness, applicability and costs), however, novel technologies that had been introduced in general clinical genetic practice (as described in the chapters above) are now also being introduced in the field of PGD and starting to replace PGH. The microsatellite markers used in PGH can be replaced by SNP micro-arrays (today, by NGS as well, see below) and, for the first time, make the test genome-wide and “universal” for all inherited familial monogenic diseases as well as chromosomal abnormalities [132,133,134]. This method will greatly eliminate the necessity for specific assay design and will therefore make PGD applicable to almost any monogenic or chromosomal disease, or combinations of these, as long as the monogenic disease is familial and a familial reference is available (next to the couple) to determine the risk haplotype. There are also some caveats to the implementation of this technique: there is a need for a SNP platform and accompanying micro-arrays for each blastomere, making a financial investment necessary. A large amount of DNA is needed for the SNP-array, therefore a whole genome amplification (WGA) is always needed prior to analysis. The timeframe of both WGA and SNP analysis is longer than the older multiplex PCR method and therefore, in some centres, IVF procedures must be amended to accommodate the genetic analysis.

Very recently, some publications showed that NGS technology can also be applied in the PGD field, using SNP analyses in the sequencing data the same way as they are used with SNP-array platforms. Features of NGS thereby can be used to analyse both multiple single gene disorders and chromosomal abnormalities at the same time, using a single platform [135,136,137]. In addition, the feasibility to perform WGS/NGS from a single cell [138] makes such approach very attractive in the field of PGD.

As with general clinical genetic diagnosis, the novel techniques put some challenges on ethical considerations (see also Section 7). In short, both SNP-array and NGS-based PGD can show chromosomal aberrations (for instance trisomy 21, deletions and insertions) that are not related to the disease for which PGD is performed. Or, both SNP-array and NGS-based PGD can be used to screen for aneuploidies in the embryos, also known as preimplantation genetic screening (PGS), however, currently there is no evidence that this is effective and efficient in increasing pregnancy rates and child health [139]. In the future, in the case of NGS, mutations may be detected in genes other than the one for which PGD is performed.

## 4. Cancer Somatic Mutation Analysis

The exploration of cancer somatic mutations has witnessed the most massive use of NGS (Table 4) and the implementation of this technology has substantially advanced our knowledge in cancer biology, through the identification of the genetic aberrations underlying tumour initiation, development and metastasis, driver genes, driver mutations and passenger mutations [140]. It also has opened the door to several clinical applications, improved patient classification, prognosis prediction, targeted treatments, drug-resistance and pharmacogenetics (see Section 5).

Different approaches have been used in these studies, either by comparing the mutation pattern observed somatically in cancer biopsies with that observed in germline DNA from the same patients (or from a healthy tissue) or by comparing with reference DNA.

One of the first reports demonstrating the potential of NGS to detect somatic alterations explored the mutation landscape in two lung cancer cell lines using the Illumina^®^ Genome Analyser and proved that NGS technology was able to overcome the shortcomings of other technologies available at that time, which were either insensitive, inaccurate or labour intensive [141]. Later that same year [142], a second publication described the sequencing and analyses of the complete exonic and regulatory DNA regions in one patient with acute myeloid leukaemia using both Illumina^®^ and Roche 454^®^ platforms. The same authors performed a similar analysis on a second acute myeloid leukaemia patient in 2009 [143] comparing the genetic mutations in tumour and matched normal skin DNA. The fact that four of the 64 mutations identified occurred in at least one additional patient (out of 188) and that somatic mutations were frequently observed in the same specific genes (like *IDH1* gene, which was mutated in more than 8% of the samples) proved the already emerging concept that few mutations in selected driver genes underlie cancer development [140] and demonstrated the technical suitability of NGS to identify such alterations. These proofs of concept were further confirmed by re-sequencing the tumour DNA of a patient in which a number of mutations present in the metastatic lesion and existing in the lobular breast tumour that arose nine years earlier were characterised [144].

Overall, these initial studies demonstrated that NGS can identify the full range of somatic alteration in cancer, including single nucleotide mutations, insertions and deletions [142], copy number variations [141], and large genomic rearrangements [145]. After 2010, there has been an exponential increase in the use of NGS in cancer somatic mutation detection and a large number of cancer types have been explored and will be described below (Table 4).

Although the rest of this Chapter will describe DNA analyses, recent studies showed the relevance of exploring the transcriptome (RNA-seq) to identify of somatic gene-fusion molecules. Such gene-fusions can lead to chimeric proteins able to suppress tumour-suppressor genes or activate oncogenes and their assessment provides an opportunity to expand the available prognostic and treatment options (reviewed by Parker et al. [224]).

### 4.1. WES and WGS: Somatic Mutation Analyses, Cancer Classifiers and Diagnosis

Large consortia and networks undertook the effort to decipher the mutation landscape in cancer. The Cancer Genome Project (Sanger Institute, UK; [225]) aims at identifying the genetic changes occurring in human cancer [145]. Although initial studies made use of traditional technology, those published after 2009 used NGS, starting with the exploration of the lung cancer cell line NCI-H209 where insights into mutational processes, gene and pathway networks were obtained [226] and followed by the analyses of myeloid leukaemia [142,143]. Over 700 cell lines [227] and more than 30 classes of different cancers have been currently explored. The Cancer Genome Project has already compiled almost five-million cancer somatic alterations through WES-NGS screenings of over 7000 primary cancers (and WGS for more than 500 samples) [228]. The project and the mutation landscapes obtained are connected with the curated “Catalogue of Somatic Mutations in Cancer” (COSMIC) a database that gathers all aberrations already described ([229]; some of these studies are reported in Table 4). The Cancer Genome Project recently explored the drug sensitivity in a large panel of cell lines [230] to help predict a drug-response and understand the mechanisms of drug-resistance. The Cancer Genome Atlas (TCGA; [231]) represents another example of a large effort dedicated to understand tumour biology and is undertaken by the U.S. Centre for Cancer Genomics and the National Human Genome Research Institute. Launched in 2005, the TCGA integrates cancer mutations and genomic germline alterations, epigenetics, transcriptomics, and more recently proteomics. It started to implement traditional approaches, which, since 2011, have been replaced by NGS for increasingly more applications ([232]; see Table 4 for some examples). Over 30 tumour types and 10000 specimens have been analysed. There exist also additional large efforts like the AURORA initiative for metastatic breast cancer [233] or the International Cancer Genome Consortium [234] that was launched to coordinate the large-scale studies across centres and countries and curates data on genomic, epigenomics, transcriptomics, mutations with carcinogenic potential, relevant for prognosis, or therapy from more than 25,000 cancers, originated from 23 different tissue types.

### 4.2. The Use of Gene-Panels in Somatic Mutation Detection: Towards Clinical Applications

After the WES/WGS studies had deciphered the genetic mutation landscape in cancer and identified driver genes associated with distinct cancer types and mutations with therapeutic implications, several reports started to use gene-panels to screen these genes (and those already known before the NGS use) for somatic mutations in cancer patients. Soon, these approaches proved useful and combined clinical applicability, cost effectiveness and the ability to identify, within the whole population of cancer cells or cell sub-clones, genetic alterations relevant to cancer initiation, with metastatic potential or conferring drug-resistance [145,228,230,235].

#### 4.2.1. Technical Validity

Several studies validated the high sensitivity and specificity of NGS compared with standards [164,215,236]). In a study assessing the mutation landscape of 79 neurological tumours, the concordances of NGS with standard methods was 98%, with a better performance of NGS in case of low quality samples [184]. It became soon clear that NGS/gene-panels are superior to single assays for a broad range of molecular oncology testing [237,238,239] and possibly in other fields. The inter-laboratory variability is very low. A 100% concordance was reported when 171 colorectal cancer patients contraindicated for EGFR therapy based on mutation screening were re-sequenced across 17 Dutch centres [240]. Similar figures were obtained in a study exploring the genome in Acute Myeloid Leukaemia samples [241].

One technical challenge for clinical use of NGS to study tumour specimens is related to the poor-quality of FFPE material, which is the only material available in most cases [242]. However, both sequencing protocols and data analyses have been adjusted to interpret FFPE data and reduce the occurrence of artefacts and false positives [243,244], resulting in comparable outcomes between FFPE and fresh-frozen specimens [245]. The combination of Ion Torrent^®^ platform/AmpliSeq^®^ technology has been used most frequently since it is well adapted to the limitations of using FFPE DNA, i.e., low input DNA, targeting short gene segments and short turnaround time [160]. Most studies have used Ion Torrent^®^ (Table 4) in combination with a 46–50-gene-panel (Cancer Hotspot Panel) that includes the most relevant oncogenes/tumour suppressor genes [161,186,196,219,237,238,239].

Illumina^®^ platforms have also been used to analyse FFPE specimens (Table 4), and although some studies reported differences between Illumina^®^ and Ion Torrent^®^ [190], other studies showed similar outcomes [158]. One recent investigation [246] compared three target enrichment and two NGS platforms: a 13-gene-panel enriched with Fluidigm Access Array followed by Illumina^®^ (MiSeq) sequencing; a 60-gene-panel enriched via Oxford Gene Technologies^®^ SureSeq Solid Tumour hybridisation followed by Illumina^®^ (MiSeq) sequencing; and a 50-gene-panel analysed by Ion Torrent^®^ AmpliSeq^®^ Cancer Hotspot Panel. The performance of FFPE DNA from eight cell lines was comparable across the platforms/protocols. Currently, various Illumina^®^ systems are routinely used in NGS analyses combining different enrichment methods TrueSeq Amplicon Cancer Panel [176], SureSelect [157,184], OncoPanel [214] and different sequencers MiSeq [157,176,213], NextSeqXT 500 [184], HiSeq [214].

Established protocols are robust for clinical use and there is constant development of novel protocols and methods, such as combinatorial probe-anchor ligation (cPAL) chemistry on arrays or the self-assembling DNA nanoballs DNBs [217,247]. Nevertheless, a certain rate of failure must be considered in mutation analyses. For instance, the Genomics and Pathology Services at Washington University School of Medicine conducted a survey on 1528 specimens from patients who were indicated by the oncologist in care for molecular testing. Both FFPE and fresh DNA were tested by NGS with Illumina^®^ platforms between 2012 and 2014 and a 20% failure mostly due to technical reasons, was reported [248].

#### 4.2.2. Clinical Utility

Clinical use of NGS/gene-panels spans from diagnostics to prognostics and from prevention to treatability (see Table 4). One important application in diagnostics is the possibility to make an accurate diagnosis using small pre-surgical biopsies. Typically, fine-needle-nodule or -lymph node aspirations are small cytological samples and, in a large proportion of the patients undergoing this procedure, a reliable diagnosis is difficult to obtain. For instance, fine-needle-aspiration of thyroid nodules results in an unclear diagnosis (intermediate cytology) in 20%–30% of the cases. The ThyroSeq gene-panel that includes driver genes and alterations frequent in thyroid tumours [198] was applied to 143 fine-needle-aspiration samples (91 retrospective and 52 prospective) with known surgical outcome (104 were benign, 39 were malignant nodules). This study showed over 90% sensitivity, specificity, accuracy with 83% of positive predictive value and 96% of negative predictive value. The same team of scientists validated and confirmed these results using an improved version of ThyroSeq gene-panel (one extra gene based on COSMIC was added) in a subsequent study on 465 consecutive fine-needle aspirate samples with intermediate diagnosis [199]. Trans-thoracic fine-needle aspiration in lung cancer [179] and endoscopic ultrasound-guided fine-needle aspiration in gastrointestinal neoplasms [177] and other primary and metastatic cancers [249] proved efficient to identify actionable and clinically relevant mutations. In line with these studies, lymph node specimens sampled via endoscopic ultrasound fine-needle aspirates could detect pathogenic alterations relevant to predict drug-resistance and to improve individualised care of rectal cancer patient [158].

Gene-panels/NGS also proved suitable in identifying mutation profiles with prognostic potential, able to predict the progression of pre-malignant into malignant lesions and to better classify tumours after surgery. Specific mutations in *GNAS* and *KRAS* could predict the progression of intraductal-papillary-mucinous-neoplasmic lesions to pancreas cancer [170], and mutations in *KRAS* have prognostic value in pancreatic ductal adenocarcinoma [168]. Genomic mutations analysed in 104 triple negative breast cancer cases highlighted the occurrence of frequent alterations in the PI3K pathway and such mutation pattern related to the clinical outcome of patients [151]. A 48-gene-panel was demonstrated effective in identifying mutations in gastric hyperplastic polyps predicting the progression through dysplasia and adenocarcinoma [176] and ultra-deep sequencing of 10 clinically actionable genes among 345 oral cavity squamous cell carcinoma resulted in mutation patterns that correlated with disease-free survival, patient prognosis and identified several actionable mutations [165].

A recent study reviewed the records of 439 patients with various types of cancers that underwent various NGS analyses at the “Center for Personalized Cancer Therapy” (La Jolla, U.S.; [250]) and found that 20% of the patients have an actionable mutation targeted by on-label drugs, and additional 50% present actionable mutations targeted by an off-label but approved drug. Wong and co-workers conducted one of the largest, prospective, multisite studies across Australia and screened 800 newly diagnosed patients with one out of over 20 cancer types (48-gene-panel). The authors demonstrated that 63% of the patients carried clinically relevant mutations, with 26% displaying a mutation with therapeutic implications [242]. A second survey on 2221 cases from the Foundation of Medicine (Foundation Medicine^®^, Cambridge, MA, USA) also concluded that actionable mutations were identified in 76% of the subjects and results were further confirmed in independent investigations [157,184,236,251,252,253,254].

The potential utility of gene-panel/NGS analyses to identify drug targets and drug-resistance mutations is also very well documented for several cancer types. A recent prospective study on 114 patients with metastatic colorectal cancer identified mutations in *KRAS*, *NRAS* and *BRAF* that are associated with resistance to anti-EGFR therapy [161]. Twenty-nine cervical cancer samples were screened with a panel comprising 226 genes and 48% of the patients displayed deleterious mutations in genes targetable with approved drugs [157]. In head and neck squamous cell carcinomas, distinct mutation profiles could identify subgroups of patients with poor outcome after adjuvant chemo radiation, as well as new potential actionable mutations [162]. Gene alterations, some of which actionable, were found after screening 76 neurosurgical brain metastases from lung cancer [181], with most frequent mutations occurring in *TP53* (over 40% of the metastases) followed by *KRAS* and *CDKN2A*. In hematologic tumours, application of gene-panels/NGS using locus-specific primer sets for immunoglobulin has prognostic value in detecting the persistence of minimal residual disease during therapy, as shown in acute lymphoblastic leukaemia [205] and multiple myeloma [204].

NGS offers also the possibility for an improved classification and for the identification of actionable mutations in neurological tumours as well as in paediatric oncology. Potential targets for therapy were identified among a panel of 130 genes in 79% of the glioblastomas, in 90% of pilocytic astrocytomas and in 36% of the medulloblastomas screened [184]. Paediatric oncology in particular presents some challenges because of the unique (and poorly characterised so far) cancer genomics. A thyroid specific gene-panel demonstrated promising to better classify thyroid tumours, with important implications for the care of the patients [199,213], and the clinical utility of NGS-based mutation discovery in paediatric patients has been recently explored within the iCat trial (see below and Table 4 [214]). Additional examples of NGS in somatic mutation analyses are reported in Table 4.

#### 4.2.3. Clinical Trials

Recently, clinical trials have been started based on the use of gene-panels/NGS. The Individualized Cancer Therapy (iCat) Study on paediatric patients (NCT01853345 [214]) enrolled 100 participants (30 years or younger) at four U.S. academic medical centres. Patients were diagnosed with high-risk, recurrent, or refractory extra cranial solid tumours between September 2012 and November 2013 and they had one-year follow-up. The study concluded that 43% of the patients had clinically relevant results through NGS analyses with 31% of the patients receiving a recommendation for individualized cancer treatment and 3% receiving a matched therapy.

The Korean NEXT1 trial assessed the mutation profile in 407 prospective metastatic cancer patients enrolled between 2013 and 2014 (281 were also checked for copy number variations). In 84% of the subjects at least one aberration was detected, and when 103 patients were matched to molecularly targeted agents, the response rate was significantly higher in the mutation-matched versus the non-matched treated group [219].

The MPACT trial (molecular profiling-based assignment of cancer therapy; NCT01827384) is the first randomized study that aims at assessing the response of patients treated with a drug that is matched to the mutation profile obtained through NGS analysis. This pilot phase II trial is currently running and recruiting patients, who are randomized to a drug matched to a somatic mutation or to a control (standard) treatment [255].

#### 4.2.4. Conclusive Remarks on Gene-Panels for Somatic Mutations Analysis

The overall clinical potential of NGS is well demonstrated. Its utility goes beyond the hype. Spreading its use will result in higher-sensitivity diagnostic methods, and will allow to better individualise treatment strategies. The long-term outcomes in terms of better survival, clinical relevant benefits for the patients and costs are being currently explored in clinical trials. In addition, one should consider that implementing these technologies in a clinical setting needs to be accompanied by easy ways to interpret the outcomes for the patients and the clinicians. Therefore, in order to further aid the clinical translation of NGS use, user-friendly clinical molecular diagnostic assays have been developed and validated. The JAX Cancer Treatment Profile™ [175,256], based on Illumina^®^, and also supported by a bioinformatics pipeline, screens 190 genes using FFPE DNA for any kind of actionable variations, small insertions, deletions and gene amplifications. The Washington University developed a number of diagnostic assays based on Illumina^®^ platforms such as the UW-OncoPlex, which covers almost 200 genes and has been validated on over 100 samples of different cancer (colon cancer, melanoma, acute myeloid leukaemia, myeloproliferative disorders, chronic myeloid leukaemia, lung cancer, gastrointestinal stromal tumour, and other neoplasms [257]). The 25 gene-panel WuCAMP has been validated on 78 cases (breast, colon, gastrointestinal, lung, cholangio and pancreatic carcinomas and hematologic tumours [258]). The ThyroSeq (already described earlier) on Ion Torrent^®^ platform has been validated for adults and paediatric oncology [198,199]. Similar to hereditary cancer syndromes, the increasing number of genes tested returns information that presents challenges in the interpretation and in the translation into clinical guidelines and practice [259,260] (see also Section 7), and a number of initiatives attempt at coping with this issue. The “Molecular Tumor Board” has been established with the goal to facilitate the interpretation of individual genetic profiles, to provide treatment recommendations and, ultimately, to increase the awareness of both clinicians and patients about these novel technologies [261]. Similarly, a recent initiative by the U.S. Personalized Health Care Committee is trying to harmonize the use of gene molecular testing and assist the pathologists and the clinical oncology communities to best use the NGS data [176].

### 4.3. Liquid Biopsy Analyses

The blood of cancer patients carries circulating tumour cells (CTC) and cell-free tumour DNA (ctDNA) that originate from either the primary tumour or from metastatic lesions and can be used as biomarkers. CTC and ctDNA are easily biopted from patients with minimal invasiveness (“liquid biopsy”) and can be used for diagnosis, prognosis, recurrence-risk prediction, or can be biopted at regular intervals to dynamically monitor disease progression, drug response and to tailor cancer care [262,263]. Especially during the process of metastasis, which is the main cause of cancer-related death, liquid biopsies can help in the early detection of tumour cells during the initial colonising process that leads to the formation of metastatic tumours [264,265]. CTC also carry information that can shed light on the process of metastasis itself. In a study on patients with colorectal cancer, 68 colorectal cancer-associated genes were screened by NGS in primary tumour, metastases and CTC. Although mutations in driver genes were present in all biopsy types, CTC contained several additional mutations that were also present in the primary lesions at sub-clonal level only, indicating the origin of tumour metastasis [266].

The first reports that proved the utility of NGS applied for liquid biopsy analysis were published in 2012. Forshew and co-workers [267] used tagged-amplicon deep sequencing to identify mutations in a small genomic region of approximately 6 Kb. Through the analysis of ctDNA in 46 plasma samples from ovarian or breast cancer patients, tumour mutations in *TP53* and *EGFR* genes were identified. Similarly, high sensitivity and specificity of NGS was demonstrated in a screening of 100 plasma samples for lung cancer patients [268]. Mutations in five cancer-related genes (*EGFR*, *KRAS*, *BRAF*, *ERBB2*, and *PI3KCA*) could be detected both in ctDNA as well as in the primary lesions.

CTC analysis by NGS was demonstrated to be a good alternative to metastatic biopsies in a study where 17 breast cancer patients were screened for mutations in 50 cancer-related genes using CTC, metastasis and primary tumour biopsies [269]. Lebofsky and co-workers [270] further extended this concept through the NGS screening of 46 genes in plasma DNA compared with metastasis from 34 patients covering 18 different tumour types. In a recent study on 377 samples from 12 patients with recurrent or progressive/metastatic bladder cancer, NGS analysis was shown to have diagnostic potential using ctDNA isolated from both plasma and urine. This was further confirmed in a larger patient group (211 patients and 20 controls) [271].

Liquid biopsies offer also information about the presence of actionable mutations. CTC extracted from the plasma of patients with a variety of cancers were screened using a 54-gene-panel/NGS and 65% of the different cancer types had detectable ctDNA aberration and most were theoretically actionable [272,273]. The assessment of mutations in *EGFR* by CTC-NGS analysis can be used to optimise pharmacologic treatment in patients with non-small cell lung cancer [274]. Specifically, ctDNA-NGS proved useful to identify mutations, amplifications and translocations in *EGFR*, *MET*, *ALK* and *ROS1* for which targeted therapies are available [275]. CtDNA-NGS analyses also proved useful to detect resistance mechanisms to tyrosine-kinase-inhibitors, such as the EGFR:T790M mutation [275]. Also Braig and co-workers [276] demonstrated that NGS analysis of liquid biopsies, applied to 46 head and neck squamous cell carcinoma patients, could identify mutations in EGFR and RAS pathways predicting resistance to the drug cetuximab. Activating mutations in the oestrogen receptor gene *ERS1* that could potentially lead to resistance to endocrine treatment were identified through a gene-panel-NGS screen of ctDNA in 48 breast cancer patients [277].

In conclusions, liquid biopsy analyses by NGS have been applied to several cancer types like hepatocellular carcinoma [278], gynaecological cancer [267], lung cancer [268,279], urinary tract cancer [271], paediatric oncology [280], gastrointestinal tumours [281] and other types, even through WES/WGS analyses [138,282]. This technique, without the need of an invasive biopsy from the diseased tissue, can help in early detection, monitoring for response to therapy, potential resistance to therapy, and can influence the choice for (alternative) therapy. It will be important to confirm these results using evidence-based approaches and clinical trials.

## 5. Pharmacogenetics

Drugs have different effects on patients concerning the level of toxicity and therapeutic effect. Several factors are known to influence treatment outcome, such as environmental factors, diet, lifestyle, disease conditions and co-medication. Increasing evidence shows that genetic variation in genes encoding drug-metabolising enzymes or other proteins within relevant drug pathways determine the individual drug response or susceptibility to adverse drug reactions (ADRs). It is estimated that these variations can account for 20% to 95% of the variability in drug response [283]. Many patients experience ADRs, part of these even lead to hospital admission and mortality, indicating that there is not only considerable harm but also a large economic burden. Indeed, several studies show that costs associated with these events during hospitalisation are high. Specifically, a study performed in a 5-month period in The Netherlands showed that the majority of hospital admissions due to health care related adverse events in an internal medicine department (emergency department) are medication related (43.5%) and 26.9% chemotherapy related [284]. Besides, part of the patients does not respond adequately to medications. It is estimated that mean efficacy of drug treatment is only about 50% and the current trial-and-error approach in pharmacotherapy is far from ideal. A relation with treatment outcome has been established for several genetic variants, and the study of these relations is called pharmacogenetics (PGx). The term pharmacogenomics refers to similar explorations used in a genome-wide approach, i.e., studies that investigate how all genes (the genome) can influence drug response. However, in literature, these terms are often used interchangeably.

Many studies focused on PGx for anticancer agents, mainly because of the severity and, sometimes, fatal toxicities of chemotherapeutics in combination with low efficacy of treatment. Until now, methods used in PGx mainly involve a candidate gene approach. This approach focuses on specific genes known to have relations to certain medication, involved in specific pathways or drug-resistance mechanisms. In oncology, both germline DNA and tumour DNA (somatic) can be explored (see also Section 3 and Section 4). In some types of cancer, the tumour genome can be used to identify certain biomarkers, predictive or prognostic in nature, which can be used for targeted therapy [285,286]. Certain drugs for example are not effective or may become toxic when cancer cells contain specific mutations (see also Section 4). One important germline PGx gene for cancer related medication is *DPYD* (gene encoding for the dihydropyrimidine dehydrogenase: DPD) [287] involved in the degradation of fluoropyrimidines, which are the main chemotherapeutic agents used in many types of cancer: 5-fluorouracil, (5-FU), capecitabine, and tegafur. DPD dysfunction leads to an increased exposure to active metabolites, which can result in severe or even fatal toxicity [288]. An overview of some additional relevant genes used onco-PGx testing has been published recently by Pesenti and co-workers [289], who discuss the importance of germline mutations and variants in onco-PGx. Overall, patients who are treated based on their PGx profile are less prone to develop side effects and can be treated more effectively.

The NGS approach for PGx can involve the analysis of the entire exome (WES) or the entire genome (WGS). WGS has the main advantage of encompassing also the non-coding regions, e.g., involved in regulation of transcription or affecting splicing, which is not present in WES data. For some PGx genes it is known that these regions are of interest [290]. For example, some studies identified variants initially thought to be causative of a specific feature or ADR, but such characteristic was subsequently associated with an intronic variant instead. This is the case for the deep-intronic variant in the *DPYD* gene, c.1129-5923C>G, which was in perfect linkage with the c.1236G>A variant previously associated with 5-FU toxicity [291]. As stated in the previous Chapters, the major current limitation of WGS/NGS is related to the complex data analysis and the (still) relatively high costs.

PGx explorations based on NGS/WES identified several novel variants potentially able to modify the therapeutic efficacy of drugs, e.g., clopidogrel [292], escitalopram [293], clozapine [294] and paclitaxel [295]. NGS can reveal both known and rare PGx variants in one test and is therefore a suitable method for the identification of novel relevant variants related to the use of certain drugs.

WGS studies in PGx research are relatively few [296,297], but it is very likely they will increase in the near future, for the several reasons given already for other applications (decreasing costs, improved bioinformatics). Since PGx in diagnostics has mainly focussed on one or a few genes related to the use of one particular drug or several drugs metabolised via the same pathway, it is likely that initially WES/WGS for PGx will be accompanied by in silico selection of ROIs. Filtering NGS data to restrict the analyses to known variants for which guidelines exist has recently been used by Yang et al. [298], who explored PGx genotypes for 13 genes with guidelines from the Clinical Pharmacogenetics Implementation Consortium (CPIC; see below). The authors compared the use of DMET- (Drug Metabolizing Enzymes and Transporters)-array based approach with WES/WGS data from the same patients and identified discordant genotypes in only a few samples at specific loci. Reasons for this were, for example, (paralogous) mapping problems or other issues related to the fact that some non-coding parts of the genome were missed in WES. However, these analyses were performed using standard analytic pipelines, which means that there is still room for improvement and increase coverage of challenging positions in the genome [298].

Before NGS for PGx will become routine in a clinical setting, additional validation is required. Guidelines already exist in PGx (recently reviewed [289]): the Pharmacogenomics Knowledgebase (PharmGKB [290]) is a resource that includes information concerning guidelines from the Clinical Pharmacogenetics Implementation (CPIC [299]) and the Dutch Pharmacogenetics Working Group (DPWG [300]). Although these guidelines try to tackle the problems related to the use of NGS in PGx (see also Section 7), the shift from candidate-gene to NGS/several-gene or even WES/WGS approaches is accompanied by the discovery of several novel variants with potential PGx implications, but for which it is often difficult to determine their influence on treatment outcome. In case of investigating the exome or genome beyond the confined list of actionable PGx variants in diagnostics, similar problems common to other fields like hereditary cancer syndrome (Section 3) occur and concern the identification of VUS and the definition of their real clinical value, diagnostic and drug-response relevance. Not only the identification of incidental findings is a problem, but also the fact that without solid evidence regarding the functional effect of a VUS it is difficult to provide a dose guideline, even within a known PGx gene. This problem will be even more important in WGS due to the higher chance of finding VUS. One possible option to solve such problem would be to initially focus on the actionable PGx genes and, if a patient experiences clear ADRs pointing to a specific drug or pathway, one may continue performing a more extended analysis (still focussed on PGx relevant pathways). However, interpretation of additional variants can still be problematic and the latter is certainly not applicable to pre-emptive PGx.

Despite these technical and clinical issues, WES/WGS information will offer clear and unique advantages. For instance, PGx information about a high number of genes at the same time may be more useful than single gene as people often use different types of medications. Moreover, this information remains available throughout the entire lifetime of a patient and will be readily accessible for future use of medications. Also in practice, the need for more PGx information, i.e., information regarding different types of medication (and therefore different genes) seems to increase in patients as well as referring physicians.

In conclusion, NGS applied to PGx shows important added values. The awareness and interpretation by the referring physicians and by the patients of the analytical PGx outcomes is of crucial importance for making this approach feasible and successful. Altogether, some issues still need to be solved and parts of the procedures improved before NGS will be the first method of choice in PGx [298,301]. Once these are solved, NGS in the field of PGx will very likely become an integral part of personalised health care.

## 6. Other Applications and Future Directions

In principle, any DNA or RNA molecule can be analysed by NGS. For instance, DNA fragments obtained after chromatin-immunoprecipitation (ChIP-seq) identify genome-wide the chromatin binding sites of a transcription factor (the so called cistrome). In hormone dependent breast cancer, ChIP-seq was used to characterise the cistrome of the oestrogen receptor, and it was demonstrated that different receptor cistromes could predict patient prognosis and were associated with the development of therapeutic resistance to endocrine drugs [302]. Similar observations were recently reported for endometrial cancer [303].

DNA methylation has been assessed by ChIP-seq using chromatin methyl-binding-domain immunoprecipitation [304], or by applying NGS directly to bisulphite-treated DNA, as recently used to characterise the methylation profile in breast cancer patients and controls [305]. Additional epigenetic events like microRNA expression can be characterised by NGS [306] as also demonstrated by The Cancer Genome Atlas [225]. Furthermore, a protocol modification in the preparation of RNA-seq samples, allows sequencing transcripts that are being translated at a certain moment in the cell [307], which is named translatome. Several studies have been able to extract meaningful biological information using this approach. Under cellular stress there is differential mRNA translation and molecules relevant for the stress response will be translated. King and Gerber have recently reviewed the methods available for translatome profiling [308]. NGS has been used to detect microsatellite instability [309], to study and diagnose mitochondrial diseases [310], and to detect pathogens in human biological specimens [311].

Finally, the feasibility of NGS analysis (WES or WGS) from a single cell is nowadays a reality [138] and this opens novel avenues in meiotic recombination of germ cells, PGD and PGS, de novo mutation rates, early evolution of cancer genomes, sub-clone and improved CTC analyses.

## 7. Limitations and Complications of NGS in Genetic Diagnostics and Ethical Considerations

A number of issues should be considered when NGS is used in genetic diagnostics, somatic mutation analysis or PGx. Once the mutation profile in a patient is characterised, the genetic aberrations have to be classified, to distinguish variants that are pathogenic from those that are not associated with the disease, and defining the clinical guidelines is always a challenge.

In the field of hereditary syndromes, there are several guidelines that assist in variant classification [22,312,313]. However, because of the incomplete penetrance of mutations involved in cancer syndromes, the identification of a pathogenic mutation in a subject and in a family does not implicate that all subjects of that family that carry the mutation will develop cancer. In addition, a mutation can confer high-risk to develop cancer at a specific site, for which counselling may be relatively trivial, but it could also be moderate- to low-risk for other cancers (Table 3). What makes the scenario even more complex is the identification of variants for which the clinical relevance is (currently) unclear (VUS). Although this complication is not unique to the use of NGS per se, and Sanger sequencing also returns VUS, it is amplified simply because when large gene-panels (or WES/WGS) are being screened, more genes and more variants are identified per patient. Re-sequencing using NGS the high-risk genes that had previously tested negative on standard methods can give good results in some cases [61,62], but in other cases, extra genes need to be analysed.

The use of gene-panels in hereditary cancer syndromes shows that about 40% of the patients tested carry a VUS [31,33,37,42,84]. The number of detected VUS increases with the number of genes tested (single gene < multiple gene-panel < WES < WGS) which makes debatable how appropriate will be the use of WES and WGS in clinical diagnostic when costs will be competitive with those of the gene-panel testing. The presence of VUS poses problems in the counselling of the patients and their family since a causal relationship between disease and the variant is unclear. Testing healthy individuals is uninformative because the presence or absence of the variant cannot be used in an effective cancer-prevention strategy, like for example preventive mastectomy or ovariectomy in case of HBOC patients, as the true cancer risk is still unclear. Several genetic, pathology societies and professionals are continuously updating the recommendation in order to parallel the increasing amount of genetic information that will be available [27,64,95,314,315]. Nevertheless, large-scale studies and segregation/functional investigations are needed to define the true cancer-risk associated with mutations in novel cancer genes.

Furthermore, as gene-panels and WES/WGS will be more frequently performed as first/standard test, increasingly more incidental, secondary or unsolicited findings will be detected. These are genetic variants that are medically relevant but not for the disease for which the patient visited the clinic [316]. The debate on the reporting of these variants to the patient is still on-going. The guideline as defined by the American College of Medical Genetics, recommends pro-active analyses and reporting of mutations of specified classes or types in a specified list of genes. This should be performed for all clinical germline exome and genome sequencing, irrespective of patient age, but excluding foetal samples. The European Society of Human Genetics [317], and the guidelines for diagnostic NGS as published by Eurogentest [318] are less strict, but local policy with respect to dissemination of secondary findings should be clear for the patient pre-testing. In all cases, informed consent is very important, meaning that the patient is educated by a health care professional about the test procedure, the benefits, limitations and the possible consequences of the test results. Based on this information the patient can make an educated and voluntary decision about having the test done or not. Assessment of incidental findings could be performed by an independent expert panel to determine their clinical relevance and penetrance, taking also into account whether it concerns a treatable, preventable health problem or not.

This increasing amount of detailed genetic information generated by novel technologies, leads to challenges in ethical considerations with regard to the principles of reproductive autonomy [310]. Arrays and NGS will allow to simultaneously test IVF and PGD embryos for multiple mutations and conditions and this will raise several issues such as making adequate embryo transfer decisions, influence of serious and non-serious conditions, the possible conflicts between clinicians and future parents, and the rights of the future child [319,320].

Somatic cancer mutation screening can identify activating mutations and variants with actionable or theranostic potential. Also in this case, however, the distinction between passenger and driver mutations and the implementation of the NGS data in patient care is not straightforward [259,260]. Most mutations with putative clinical relevance are rare and their utility cannot be assessed in typical Phase III clinical trials. A pragmatic approach can be not to focus on each disease/each mutation subset but to use rather the so called basket trial, where different tumour types having the same genetic alteration are included and through a specific study design “baskets” are enriched for patients with a specific tumour type or histology [260]. In addition to these strategies and innovative study designs, committees are established to make treatment recommendations like the “Molecular Tumor Board” [253] and the US Personalized Health Care Committee [194].

In the field of PGx there exist similar complication in relation to the occurrence and interpretation of VUS as described earlier. The Netherlands is one of the few countries with existing guidelines for PGx and in particular the Dutch Pharmacogenetics Working Group (DPWG [300] from the Royal Dutch Pharmacists Association, KNMP [321]) has developed dosing guidelines for about 80 gene-medication combinations. Similar guidelines have been formulated by other authorities as well like the Clinical Pharmacogenetics Implementation Consortium (CPIC [299]).

Despite these limitations and complications, some studies have assessed patient reactions to the use of NGS in clinic, and satisfaction for inherited cancer syndrome diagnostics is high and associated with low distress, when accompanied by proper counselling [314]. In addition, total health care costs, life-years gained, and quality-adjusted life-years associated with the use of a NGS/gene-panel provide meaningful clinical benefits in a cost-effective manner per quality-adjusted life-years threshold [322]. The evaluation of the socio-economic aspects of NGS in genetic diagnostics will differ between countries in relation to the health care system. In a recent patterns-of-care study conducted in the U.S. [323] that compared demographic, socioeconomic, and clinical information among patients with inherited colorectal cancer or polyposis syndromes who were tested using gene-panel/NGS or with the reference method, it was concluded that NGS gene-panel testing is more frequently performed among insured subjects.

We can envisage that initial positive performance of NGS will further improve in the future, because of the increasing availability of large datasets with patient clinical and genetic information that will allow making better prediction of the effect, penetrance, classification and the implications for PGx of novel variants and current VUS [324,325].

## 8. Conclusive Remarks

NGS has brought unprecedented advances in understanding the biology of diseases, with important clinical implications. Genetic screening of germline and somatic DNA mutations (either from tumour specimens or liquid biopsies) and RNA analyses can importantly aid patient care. The genetic services offered through the public or private sector are easily accessible and thorough and allow setting up personalised genetic screenings (from a few to several genes) and clinical options, an important step towards individualised medicine.

Technical and bioinformatical advances make the NGS technology increasingly more powerful. It is crucial that these progresses are accompanied by increasing awareness of its strong potential by physicians and patients. It is also of fundamental importance that the progress is paralleled by strict monitoring the use of these technologies in relation to ethical issues and to keep the balance between hope and hype.

## Figures and Tables

**Table 1:** Some of the gene-panels developed and clinically in use for Hereditary Breast/Ovarian Cancer (HBOC) and Colorectal Cancer/Lynch syndromes. Major technical characteristics of the panels are reported together with some relevant references.

Developer	Name	ROIs ^1^DNA Capture Method and FeaturesKind of Study/Purpose	No. Genes ^2^	Reference
Mayo Clinic	No specific name was given	- ROIs: exonic regions, 272 Kb - Enrichment: a. solid-phase (NimbleGen 385K)—PCR (ligation mediated) amplification; b. *Bam*H1 digestion of library for Illumina sequencing (fragmentation) - Sequencer: a. GS-FLX Roche 454 (average depth: 30 reads/bp); b. Illumina (Genome Analyzer II; average depth: 266 reads/bp) - Preclinical: 5 patients with Lynch syndrome	22	[23]
University of Washington	BROCA	- ROIs: exons, introns, 10 Kb at the 3′ and 5′s genomic region, 1 Mb - Enrichment: oligo based hybridisation in solution - a. Sequencer: Illumina (Genome Analyzer IIX; depth > 1200 reads/bp) - Preclinical: 20 women with HBOC	21	[24]
		- b. Sequencer: Illumina (Genome Analyzer IIX; depth: 449 reads/bp) - Preclinical: 360 women with ovarian cancer	21	[25]
		FROM 2012—Clinical use - ROIs: variable - Enrichment: oligo based hybridisation in solution - Sequencer: Illumina (HiSeq2000; depth: 320–1000)	Up to 66	[26]
	ColoSeq	- ROIs: exons, introns, 10 Kb at the 3′ and 5′s genomic region, 1.1 Mb - Enrichment: oligo based hybridisation in solution - Sequencer: Illumina (HiSeq2000; depth: 320) - Preclinical: 82 DNA specimens, 7 genes (209 Kb) analysed	31 (7)	[27]
		FROM 2012—Clinical use - Sequencer: Illumina (HiSeq2000; depth: 320–1000)		[28]
François Baclesse, France	(Custom Design Castera et al.)	- ROIs: exons, introns - Enrichment: oligo based hybridisation in solution - Sequencer: Illumina (Genome Analyzer IIX) - Clinical utility: 708 suspected HBOC patients	16–24	[29]
Memorial Sloan-Kettering Cancer Center	(Custom design)	- ROIs: exons - Enrichment: RainDance technology - Sequencer: Illumina (MiSeq; depth: 1765×) - Feasibility: 30 patients with myeloid malignancies	28	[30]
Ambry Genetics	BreastNext, OvaNext, ColoNext, CancerNext	- ROIs: exons - Enrichment: RainDance technology - Sequencer: Illumina (HiSeq, MiSeq NGS, 2000) - Centre experience: 2079 patients	14–21	[31,32]
	ColoNext	- Centre experience: 586 patients, personal history of colorectal cancer indicated for genetic test; subject mutations status was unknown. Pathogenic mutation identified in 10% of the patients	14	[33]
	OvaNext	- Centre experience: 911 patients referred for gene testing for HBOC	21	[34]
Myriad	Multiple Hereditary Cancer Panel	- ROIs: exons - Enrichment: RainDance technology - Sequencer: Illumina (MiSeq or HiSeq2500)		[35]
		- Clinical feasibility: 1964 patients with or suspected hereditable cancer syndrome	25	[36]
		- Clinical feasibility: 1260 patients with suspected Lynch syndrome	25	[37]
		- Clinical experience: 2158 patients with suspected hereditable cancer syndrome	25	[38]
University of Tubingen		- ROIs: exons - Enrichment: enrichment by hybridisation and amplification by a. in solution HaloPlex technology (56–58 genes) b. oligo based hybridisation in solution (94 genes) - Sequencer: Illumina (MiSeq) - Clinical feasibility: 620 patients with high-risk HBOC family profile indicated for genetic testing	56–94 (10)	[39]
Trinity College Dublin		- ROIs: exons, 1.6 Mb - Enrichment: oligo based hybridisation in solution - Sequencer: Illumina (Genome Analyzer II/HiSeq2000; depth: 89×) - Clinical feasibility: 104 patients with no *BRCA1*/*2* mutation and 101 controls	320	[40]
Invitae	Various gene-panels available	- ROIs: exons, 95 Kb - Enrichment: oligo based hybridisation in solution; integrated for low-covered regions with DNA Technologies (Coral, IL, USA) xGen Lockdown probes - Sequencer: Illumina (MiSeq, HiSeq2500; depth: 450×)	42–80	[41]
	Colorectal Cancer Panel	- Clinical feasibility/experience: 1062 HBOC subjects	29	[42]
Emory Genetics Laboratory ^3^	Several gene-panels available	- ROIs: exons - Enrichment: oligo based hybridisation in solution - Sequencer: Illumina (HiSeq 2500)	19–60	[43]
GeneDX ^4^	Various gene-panels available	- ROIs: exons - Enrichment: oligo based hybridisation in solution - Sequencer: Illumina (MiSeq or HiSeq; depth: 100×)	20–32	[44]
		- Clinical experience: 8 panels applied to 10030 patients between 2013 and 2014	Up to 29	[45]
Fulgent Diagnostics ^5^		- ROIs: exons - Enrichment: oligo based hybridisation in solution - Sequencer: Illumina (MiSeq or HiSeq)	21–38	[46]
CentoGene ^6^		- ROIs: exons - Enrichment: oligo based hybridisation in solution - Sequencer: Illumina (Depth > 20×)	4–31	[47]
Qiagen (GeneReader technology)		- ROIs: exons - Enrichment: amplicon-based PCR - Sequencer: Qiagen (Ultra depth)	Up to 20	[8]

^1^ ROIs: regions of interest; in most cases, exons include few intronic nucleotides flanking each exon; ^2^ Number of genes captured and analysed; by brackets, if number of genes analysed does not correspond to captured genes (in silico definition of ROIs from larger datasets); ^3^ Emory Genetics Laboratory offers several gene-panels including: Breast and Ovarian Cancer-Sequencing and Deletion/Duplication Panel; Hereditary Cancer Syndrome; Gastrointestinal and Colorectal Cancer; High-risk Breast Cancer Panel; ^4^ GeneDX offers gene-panels including: Breast Ovarian Cancer Gene-panel; Colorectal Cancer Panel; Comprehensive Cancer; High-moderate-risk; ^5^ Fulgent Diagnostics offers panels including ColonCancer NGS Panel (21 genes) and Breast OvarianCancer NGS Panel (38 genes); ^6^ Panels offered by CentoGene include CentoColon extended (17 genes), the CentoCancer (31 genes) and CentoBreast (13 genes) panels.

**Table 2:** Genes included in the most common gene-panels used for diagnostics. Gene-panels refer to HBOC syndrome and Colorectal Cancer/Lynch syndrome.

*Risk-category*	*GENE NAME*	Breast and Ovarian Cancer											Colorectal Cancer							All Types						
		BROCA ^1^	Custom Design ^2^	BreastGene ^3^	BreastNext ^4^	HBC High-Risk ^5^	High Risk BC ^6^	CentoBreast ^7^	B/OC Gene-Panel ^5^	B/OC NGS Pan el ^8^	BOC ^6^	OvaNext ^4^	ColoSeq ^9^	ColoNext ^4^	CRC ^10^	CRC ^5^	CentoColon ^7^	CC NGS Panel ^8^	G/CRC ^6^	CancerNext ^4^	Cross-Cancer ^10^	Comprehensive ^5^	H-M Risk ^5^	myRisk ^11^	HCS ^6^	CentoCancer ^7^
High-risk genes	*APC*	◉								◉			○	◉	◉	◉	◉		◉	◉	◉	◉	◉		◉	◉
	*BMPR1A*	◉	A							◉			○	◉	◉	◉	◉	◉	◉	◉	◉	◉	◉	◉	◉	◉
	*BRCA1*	○	A	◉	◉	◉	◉	◉	◉	◉	◉	○	○						◉	◉	◉	◉	◉		◉	◉
	*BRCA2*	○	A	◉	◉	◉	◉	◉	◉	◉	◉	○	○						◉	◉	◉	◉	◉	◉	◉	◉
	*CDH1*	○	A	◉	◉	◉	◉	◉	◉	◉	◉	○	○	◉	◉	◉	◉	◉	◉	◉	◉	◉	◉	◉	◉	◉
	*CDKN2A*	◉								◉			○					◉	◉	◉	◉	◉	◉	◉	◉	◉
	*EPCAM*	○							◉	◉	◉	○	○	◉	◉	◉	◉	◉		◉	◉	◉	◉			◉
	*MLH1*	○	B						◉	◉	◉	○	○	◉	◉	◉	◉	◉	◉	◉	◉	◉	◉		◉	
	*MSH2*	○	B						◉	◉	◉	○	○	◉	◉	◉	◉	◉	◉	◉	◉	◉	◉		◉	◉
	*MSH6*	○	B						◉	◉	◉	○	○	◉	◉	◉	◉	◉	◉	◉	◉	◉	◉		◉	◉
	*MUTYH*	○			◉	◉				◉	◉	○	○	◉	◉	◉	◉	◉	◉	◉	◉	◉	◉	◉	◉	◉
	*PMS2*	○	B					◉	◉	◉	◉	○	○	◉	◉	◉	◉	◉	◉	◉	◉	◉	◉		◉	◉
	*PTEN*	○	A	◉	◉	◉	◉	◉	◉	◉	◉	○	○	◉	◉	◉	◉	◉	◉	◉	◉	◉	◉	◉	◉	◉
	*SMAD4*	◉								◉			○	◉	◉	◉	◉	◉	◉	◉	◉	◉	◉	◉	◉	◉
	*STK11*	○	A	◉		◉	◉	◉		◉	◉	○	○	◉	◉	◉	◉	◉	◉	◉	◉	◉	◉	◉	◉	◉
	*TP53*	○	B	◉	◉	◉	◉	◉	◉	◉	◉	○	○	◉	◉	◉	◉	◉	◉	◉	◉	◉	◉	◉	◉	◉
	*VHL*	◉								◉			○								◉	◉	◉		◉	
Moderate-risk genes	*ATM*	○	A	◉	◉	◉		◉	◉	◉	◉	○	○		◉	◉			◉	◉	◉	◉	◉	◉	◉	◉
	BRIP1 *	○		◉	◉	◉		◉	◉	◉	◉	○	○							◉	◉	◉	◉	◉	◉	◉
	*CHEK2*	○	A	◉	◉	◉		◉	◉	◉	◉	○	○	◉	◉	◉	◉	◉	◉	◉	◉	◉	◉	◉	◉	◉
	*MRE11A*	○	A		◉	◉				◉	◉	○	○							◉					◉	
	PALB2 **	○	A	◉	◉	◉	◉	◉	◉	◉	◉	○	○							◉	◉	◉	◉	◉	◉	◉
	*POLD1*	◉											◉	◉	◉	◉	◉		◉	◉	◉	◉		◉	◉	◉
	*POLE*	◉											◉	◉	◉	◉	◉			◉	◉	◉		◉		◉
	*AXIN2*	◉								◉			◉		◉	◉		◉			◉	◉				
	*BARD1*	○	A		◉	◉		◉	◉	◉		○	○							◉	◉	◉		◉	◉	◉
	*CDK4*	◉								◉			○							◉		◉		◉	◉	◉
	*FANCC*					◉			◉	◉												◉				
	*NBN*	○			◉	◉		◉	◉	◉	◉	○	○							◉	◉	◉		◉	◉	◉
	*RAD51C*	○	A		◉	◉		◉	◉	◉	◉	○	○							◉	◉	◉	◉	◉	◉	◉
	*RAD51D*	◉	B		◉	◉			◉	◉	◉	◉								◉	◉	◉	◉	◉	◉	◉
	*XRCC2*	◉	C			◉			◉	◉	◉	◉										◉			◉	
Low-risk	*AIP*																								◉	
	*AKT*	◉				◉							◉													
	*ALK*																								◉	
	*ATR*	◉								◉																
	*BAP1*	◉	B							◉			○												◉	
Low-risk genes	*BLM*									◉					◉				◉						◉	◉
	*BUB1B*												○		◉			◉							◉	
	*CDC77*																								◉	
	*CTNNA1*	◉											◉													
	*CTNNB1*									◉																
	*DICER*																				◉					
	*EXO1*																	◉								
	*FAM175A*	◉				◉																				
	*FH*	◉																							◉	◉
	*FLCN*	◉													◉			◉							◉	◉
	*GALNT12*	◉											◉		◉			◉								
	*GEN1*	◉																								
	*GPC3*	◉																							◉	
	*GREM1*	◉											◉	◉	◉	◉				◉	◉	◉		◉		
	*HOXB13*	◉								◉											◉					
	*KIT*												○								◉					
	*MAX*																				◉				◉	
	*MEN1*	◉																			◉				◉	◉
	*MET*	◉																							◉	
	*MGMT*																								◉	
	*MLH3*		B												◉											
	*NBS1*		A																							
	*NF1*				◉	◉						◉								◉	◉					
	*NF2*																								◉	
	*NTHL1*	◉											◉				◉									◉
	*PALLD*	◉																								
	*PDGFRA*												◉								◉					
	*PHOX2B*																								◉	
	*PIK3CA*	◉				◉							◉													
	*PMS1*	○	B					◉										◉								
	*PRKAR1A*																								◉	
	*PRSS1*	◉																								
	*PTCH1*	◉																							◉	
	*RAD50*	○	A		◉	◉				◉	◉	○	○							◉	◉				◉	
	*RAD51*		C							◉																
	*RAD51B*	◉	C																							
	*RET*	◉											○												◉	
	*RINT1*	◉				◉																				
	*SDHA*																				◉					
	*SDHAF2*																								◉	
	*RINT1*	◉				◉																				
	*SDHA*																				◉					
	*SDHAF2*																								◉	
	*SDHB*	◉				◉															◉				◉	
	*SDHC*	◉																			◉				◉	
	*SDHD*	◉				◉															◉				◉	
	*SMARCA4*	◉									◉									◉	◉				◉	
	*SMARCB1*																								◉	
	*SUFU*																								◉	
	*TMEM127*																								◉	
	*TSC1*																				◉				◉	
	*TSC2*																				◉				◉	
	*XRCC3*		C							◉																

^1^ BROCA: ○ = 2010; ◉ = 2016. BROCA 2016 also comprises: *PDGFRA*, *MITF*, *FANCM*, *POT1*, *RB1 RECQL*, *RSP20*, *SLX4* [26]; ^2^ this custom designed gene-panel (Custom Design by Castera et al. [29]) has three variants: A = genes captured in three variants; B = genes captured by two variants; C = genes captured by one panel; ^3^ BreastGene, Breast Health UK [63]; ^4^ BreastNext, OvaNext (○ = 2013; ◉ = 2016), ColoNext and CancerNext, Ambry Genetics [32]; ^5^ Hereditary Breast Cancer High-Risk Panel (HBC High-Risk), Breast Ovarian Cancer gene-panel (B/OC gene-panel), Colorectal Cancer Panel (CRC), Comprehensive Cancer panel, High-Moderate-risk Panel (H-M Risk), GeneDX [44]; ^6^ High-risk Breast Cancer Panel (High-risk BC), Breast and Ovarian Cancer: Sequencing and Deletion/Duplication Panel (BOC), Gastrointestinal and Colorectal Cancer: Sequencing Panel (G/CRC), Hereditary Cancer Syndrome: Sequencing Panel (HCS), Emory Genetics [43]; ^7^ CentoBreast panel, CentoColon panel, CentoCancer panel, CentoGene [47]; ^8^ Breast Ovarian Cancer NGS Panel (/OC NGS Panel), ColonCancer NGS Panel (CC NGS Panel), Fulgent Diagnostics [46]; ^9^ ColoSeq: ○ = 2010; ◉ = 2016. ColSeq 2016 also comprises: *PDGFRA* [28]; ^10^ Colorectal Cancer Panel (CRC), Cross-Cancer Panel, Invitae [41]; ^11^ Myriad Genetics [35]; * *BRIP1* = *FANKJ*; ** *PALB2* = *FANCN*.

**Table 3:** Overview of the most common cancer syndromes. The high-risk genes are indicated and also the lifetime cancer risks conferred for distinct sites. This table is not an extensive and comprehensive review of the literature about cancer syndromes, which is outside the scope of this study.

Cancer Syndrome	Site at High-Risk of Cancer	Gene Mutated	Life-Time Risk of Cancer (%) Per Site by 70 (* 80 or ^##^ 50) Years						References
			Breast	Endometrium	Ovary	Colon-Rectum	Prostate	Other Sites	
**General Population**	**Non Applicable**		**7.3**	**1.6**	**0.7**	**1.9**	**8.2**	**2.6–0.1**	[63,64,71,72,73]
- Hereditary breast and ovarian cancer syndrome (HBOC)	Breast, Ovary Pancreas, Prostate	*BRCA1*	46–87		39–63		Up to 16		[63,64,71,72,73]
		*BRCA2*	43–84 ^#^		16–27		20	Pancreas: 7 *	[63,64,71,72,73]
	Breast, Pancreas	*PALB2*	20						[63,64]
	Breast	*RAD51A*							[74]
	Ovary, Breast	*RAD51C*							[75]
	Breast	*BARD1*							
	Ovary, Breast	*BRIP1*	20						[63,64]
- Lynch syndrome	Ovary, Colon Rectum Endometrial Pancreas, Stomach	*MLH1*		25–60	4–12	52–82		Stomach: 6–13; Small bowel: 3–6	[76]
		*MSH2*		25–61	4–13	52–83		Stomach: 6–13 Small bowel: 3–6	[76]
	Ovary, Colon Rectum Endometrium	*MSH6*		16–71		10–69			[76]
		*PMS2*		15–20					[76]
- Ataxia-telangiectasia	Breast, Pancreas Risk of Leukaemia Risk of Lymphoma	*ATM*							
- Hereditary breast and colorectal cancer	Breast, Colon Rectum	*CHEK2*	25						[63,64]
- Cowden syndrome - PTEN hammartoma tumour syndrome - Bannayan–Riley–Ruvalcaba syndrome	Breast, Colon Rectum Endometrium Other sites Risk of Melanoma	*PTEN*	77–85	19–28		9–16		Melanoma: up to 6 Kidney: 15–35 Thyroid 21–38	
- Familial adenomatousus polyposis - Syndrome attenuated familial - Adenomatous polyposis gardner syndrome	Colon, Rectum Pancreas, Stomach	*APC*		Up to 15		93 ^##^		Brain	[76]
- Hereditary diffuse gastric cancer	Breast, Stomach	*CDH1*	39–52 *					Stomach: 56–83 *	[63,64,77]
- Juvenile polyposis syndrome	Colon, rectum Stomach	*BMPR1*						Stomach: up to 20	[76]
- Juvenile polyposis syndrome - Hereditary haemorrhagic telangiectasia	Colon, Rectum, Stomach, other sites	*SMAD4*				40–50 *		Stomach: up to 20 *	[76]
- Li Fraumeni syndrome	Overall cancer risk at young age	*TP53*							[63]
- Melanoma-pancreatic cancer syndrome - Melanoma cancer syndrome	Pancreas Risk of Melanoma	*CDKN2A* ^1^						Pancreas: up to 17 Melanoma: 28–76 *	[78]
- MUTYH-associated polyposis syndrome - MUTYH-associated colon cancer risk	Colon, Rectum	*MUTHY*				3–10 *			[76]
- Peutz Jeghers syndromes	Breast, Colon Rectum Endometrium Pancreas, Stomach Ovary	*STK11*	45–50	9	18–21	39		Cervix: 10 Stomach: 29 Pancreas 11–36 Lung: 15–17 Small bowel: 13	[79]
- Retinoblastoma	Retinoblastoma	*RB*	26 *						[64]
- Hereditary mixed polyposis syndrome	Colon, Rectum	*SCG5 GREM1*							[80]
- Hereditary ovarian cancer risk	Ovary	*RAD51D*							[81]
- Melanoma-pancreatic cancer syndrome - Melanoma cancer syndrome	Pancreas, risk of Melanoma	*CDK4*							
- Nijmegen breakage syndrome	Breast, Prostate	*NBN*							
- Neurofibromatosis	Risk of sarcomas	*NF1*							
- Oligodontia-colorectal cancer syndrome	Colon, rectum	*AXIN2*							[82]
- Multiple endocrine neoplasia	Parathyroid gland, Pancreas Pituitary gland	*MEN1*							[83]
- Polymerase proofreading-associated syndrome	Colon, Rectum	*POLE*, *POLD1*							
- Von Hippel-Lindau syndrome	Kidney, Pancreas, Genital tract	*VHL*							
- Turcot syndrome	Brain	*APC*, *MLH1*, *PMS2*							

^1^ *CDKN2A* encodes for p16INK4a and p14ARF; ^#^ 7% lifetime risk for male breast cancer; ^##^ risk by age 50 years; * risk by age 80 years.

**Table 4:** Examples of NGS studies exploring the somatic mutation profile in cancer. Major features and study outcome are indicated.

Disease	Cases	Somatic Mutation-Other Analyses*	Design and Samples (FFPE **) Sequencer Aim/Project/Trial	Reference
**Breast cancer**				
Breast Cancer	15	WES	- Samples: primary tumours - Sequencer: Illumina - Project: cosmic cancer genome project	[146]
	510	WES -RNA-seq -miRNA -methylation	- Samples: frozen samples from 507 patients (invasive disease) with no prior treatment and with companion normal DNA (adjacent tissue, blood) - Sequencer: Illumina - Project: TCGA	[147]
	21	WES	- Samples: various BC (oestrogen-receptor-positive, HER2-positive, BRCA2-positive, triple negative, BRCA1-positive) - Sequencer: Illumina - Project: cancer genome project	[148]
	100	WES	- Samples: primary BC, 79 oestrogen-receptor-positive/21 negative - Sequencer: Illumina - Project: cancer genome project	[149]
Invasive lobular Breast Cancer	127	WES	- Samples: Frozen tumours (817 BC in total)/matched normal - Sequencer: Illumina - Project: TCGA	[150]
Triple Negative Breast Cancer	104	44-gene-panel	- Samples: FFPE specimens from archival triple negative tumours - Sequencer: Ion Torrent - Aim: diagnosis/classification of triple negative BC	[151]
**Gynaecological cancer**				
Endometrial Cancer	13	WES	- Samples: tumour/matched normal DNA, frozen specimens - Sequencer: SOLiD V3.0—Illumina - Aim: driver genes (including *ARID1A*, PI3K pathway)	[152]
	248	WES (107 WGS) -RNA-seq -miRNA -methylation	- Samples: tumour/germline - Sequencer: Illumina - Project: TCGA	[153]
Ovarian Cancer	9	50-gene-panel	- Samples: FFPE from high grade tumour/10 normal ovary - Sequencer: Ion Torrent - Aim: proof of concept. *TP53* frequently mutated	[154]
	316	WES	- Sample: stage-II–IV high-grade serous samples/normal DNA - Sequencer: Illumina - Project: TCGA	[155]
Mucinous Ovarian Tumours	69	50-gene-panel	- Samples: FFPE from 37 tumours and 26 border line tumours - Sequencer: Ion Torrent - Aim: prognosis and actionable mutations (*KRAS*)	[156]
Cervical Cancer	29	226-gene-panel	- Samples: 25 squamous cell, 4 adenocarcinoma and 7 normal cervix - Sequencer: Illumina - Aim: actionable mutations (*PIK3CA*, *KRAS*, *FBXW7*)	[157]
**Colorectal cancer**				
Rectal Cancer	102	50-gene-panel	- Samples: fine-needle aspiration and lymph node cytology - Sequencer: Ion Torrent - Aim: feasibility, drug-resistance, theranostics	[158]
Colorectal Cancer	276	WES (97 WGS) -RNA-seq -miRNA -methylation	- Samples: tumour/normal pair - Sequencer: Illumina - Project: TCGA	[159]
Colorectal Cancer	22	46-gene-panel	- Samples: FFPE, microdissected tumour/stroma surrounding the tumour - Sequencer: Ion Torrent - Aim: validation, performance, false positive rates	[160]
	114	50-gene-panel	- Samples: prospective metastatic samples - Sequencer: Ion Torrent - Aim: drug-resistance mutations (ERGR therapy)	[161]
**Head and Neck Cancer**				
Squamous Cell Carcinomas	208	45-gene-panel	- Samples: FFPE, locally advanced tumours, treated with adjuvant care - Sequencer: Ion Torrent - Aim: drug-resistance, actionable mutations (*TP53*, PI3K path), prognosis	[162]
	279	WES (29 WGS) -RNA-seq -miRNA -methylation	- Samples: tumours (172 oral cavity, 33 oropharynx, 72 laryngeal sites) with known HPV status/normal DNA - Sequencer: Illumina - Project: TCGA	[163]
Oropharyngeal Squamous Cell Carcinoma	8	46-gene-panel	- Samples: FFPE specimens, 4 HPV-positive and 4 HPV-negative - Sequencer: Ion Torrent - Aim: proof of concept, therapeutic and actionable targets	[164]
Oral Cavity Squamous Cell Carcinoma	345	10-gene-panel Ultra-deep seq	- Samples: retrospective node positive patient FFPE specimens - Sequencer: Ion Torrent - Aim: prognosis and target for drugs	[165]
**Digestive system: stomach, salivary glands, pancreas**				
Gastric Cancer	15	50-gene-panel	- Samples: retrospective FFPE, high-grade intraepithelial-neoplasia (IP) associated cancer - Sequencer: Ion Torrent - Aim: marker for progression of IP to cancer	[166]
Gastric Adenocarc. ^1^	238	45-gene-panel	- Samples: retrospective FFPE - Sequencer: Ion Torrent - Aim: feasibility, actionable mutations	[167]
Pancreatic Ductal Adenocarc. ^1^	73	65-gene-panel	- Samples: fresh-frozen tissues from surgical resection samples - Sequencer: (anchored multiplex PCR)—Illumina - Aim: prognosis (KRAS-G12V associated with poor survival)	[168]
Pancreatic Adenocar. ^1^	13	WES	- Samples: metastases from patients with multiple tumour lesions - Sequencer: Illumina - Project: cancer genome project	[169]
Pancreas Cancer	38	275-gene-panel	- Samples: retrospective FFPE, intraductal-papillary-mucinous-neoplasms (IPMN)-associated invasive cancer (microdissected) - Sequencer: Illumina - Aim: markers for progression of IPMN to cancer	[170]
Salivary Duct Carcinoma	37	50-gene-panel	- Samples: retrospective FFPE - Sequencer: Ion Torrent - Aim: diagnosis, actionable mutation (*PIK3CA*, *ERBB2*)	[171]
Gastric Cancer	89	50-gene-panel	- Samples: retrospective FFPE tumour of patients with metastasis - Sequencer: Ion Torrent - Aim: NGS validation, feasibility, frequent mutations in *TP53* (28%)	[172]
Gastric Adenocarc. ^1^	295	WES (107 WGS) -RNA-seq -miRNA -methylation	- Samples: tumour/germline - Sequencer: Illumina - Project: TCGA	[173]
Salivary Epithelial-Myo Epithelial Carcinoma	17	50-gene-panel -RNA-seq	- Samples: FFPE, tumours and 6 unmatched normal salivary glands - Sequencer: Ion Torrent - Aim: actionable mutations	[174]
Gastric Adenocarc. ^1^	167	46-gene-panel	- Samples: retrospective FFPE, 92 gastroesophageal junction and 75 lower stomach lesions - Sequencer: Ion Torrent - Aim: classification (gastroesophageal junction versus stomach)	[175]
Gastric Cancer	8	48-gene-panel	- Samples: gastric hyperplastic polyps (GHP)/different grades of dysplasia - Sequencer: Illumina - Aim: markers for progression of GHP to cancer, mutation profile (*TP53*)	[176]
GastricGastro-Intestinal Stromal Tumours	20	50-gene-panel	- Samples: retrospective endoscopic ultrasound-guided fine-needle aspiration specimens - Sequencer: Ion Torrent - Aim: feasibility, diagnosis	[177]
**Non-Small-Lung-Cancer**				
Squamous Lung Cancer	178	WES (19 WGS) -RNA-seq -miRNA -methylation	- Samples: frozen untreated stage I–IV/normal DNA - Sequencer: Illumina - Project: TCGA	[178]
Lung Adenocarc. ^1^	38	22-gene-panel	- Sample: retrospective trans-thoracic fine-needle aspiration cytology - Sequencer: Ion Torrent - Aim: proof of concept/actionable mutations	[179]
	230	WES (93 WGS) -RNA-seq -miRNA -methylation	- Samples: tumour (untreated)/normal - Sequencer: Illumina - Project: TCGA	[180]
	76	48-gene-panel	- Samples: FFPE neurosurgical brain metastasis analyses - Sequencer: Illumina - Aim: feasibility, actionable mutations, *TP53* frequently mutated (46%)	[181]
Lung Cancer	183	WES (23 WGS)	- Samples: tumour/normal - Sequencer: Illumina - Project: TCGA	[182]
Non-Small Cell Lung Cancer	209	23-gene-panel	- Samples: retrospective FFPE tumour specimens - Sequencer: Illumina - Aim: feasibility, diagnostic yield, actionable mutations (*KRAS*, *EGFR*)	[183]
**Neurological Cancers**				
Brain Tumour	150	130-gene-panel some introns and promoters	- Samples: FFPE, 79 retrospective (known mutations); glioblastomas (*n* = 47), pilocytic astrocytomas (*n* = 10), medulloblastomas (*n* = 14) - Sequencer: Illumina - Aim: validation NGS compared with standard, feasibility	[184]
Glioma	820	WES (42 WGS) -RNA-seq -miRNA -methylation	- Samples: frozen tumours (diffuse grade II-III-IV gliomas) - Sequencer: Illumina - Project: TCGA	[185]
Glioma	121	20-gene-panel	- Samples: retrospective cases - Sequencer: Ion Torrent - Aim: validation, histological and molecular classification	[186]
Diffuse Lower-Grade Gliomas	289	WES (73 WGS) -RNA-seq -miRNA -methylation	- Samples: 100 astrocytomas, 77 oligoastrocytomas, and 116 oligodendrogliomas - Sequencer: Illumina - Project: TCGA	[187]
Glioblastoma	291	WES (163 WGS) -RNA-seq -miRNA -methylation	- Sample: tumour/germline - Sequencer: Illumina - Project: TCGA	[188]
	44	50-gene-panel	- Samples: Fresh-frozen (Australian Genomics and Clinical Outcome of Glioma Biospecimen Resource) - Sequencer: Ion Torrent - Aim: alternative treatment options	[189]
	3	WES and 409-gene-panel	- Samples: primary, recurrent tumour, blood DNA - Sequencer: Illumina (WES)/Ion Torrent (gene-panel) - Aim: compare NGS platforms, proof of concept	[190]
**Kidneys and urinary system**				
Clear Cell Renal Carcinoma	417	WES -RNA-seq -miRNA -methylation	- Samples: Frozen tumour/matched normal kidney-blood DNA - Sequencer: Illumina - Project: TCGA	[191]
Papillary Renal-Cell Carcinoma	157	WES	- Samples: 75 type 1, 60 type 2 and 26 non-classified tumours - Sequencer: Illumina - Project: TCGA	[192]
Urothelial Bladder Carcinoma	130	WES (18 WGS)	- Samples: frozen tumours/matched blood or normal tissue - Sequencer: Illumina - Project: TCGA	[193]
Chromophobe Renal Cell Carcinoma	66	WES (50 WGS)	- Sample: tumour/germline - Sequencer: Illumina - Project: TCGA	[194]
Renal Cell Carcinoma	10	275-gene-panel	- Samples: succinate dehydrogenase negative FFPE archive samples - Sequencer: Illumina - Aim: classification	[195]
	32	50-gene-panel	- Samples: Retrospective FFPE; 22 metastatic; treated with at least one tyrosine kinase or mTOR inhibitor - Sequencer: Ion Torrent - Aim: associations between histotype, mutation and response to therapy	[196]
**Thyroid cancer**				
Papillary Thyroid Carcinoma	402	WES -RNA-seq -miRNA -methylation	- Samples: tumour/germline - Sequencer: Illumina - Project: TCGA	[197]
Thyroid Cancer	143	13 genes (exons) and 42 gene fusions	- Samples: nodule fine-needle aspiration (known surgical outcome) - Sequencer: Ion Torrent - Aim: feasibility to make a diagnosis	[198]
Thyroid Cancer	465	14 genes (exons) and 42 gene fusions	- Samples: frozen fine-needle aspiration of thyroid nodules with intermediate cytology (i.e., atypia or follicular lesions with undetermined significance); 98 samples had a definitive classification - Sequencer: Ion Torrent - Aim: diagnosis, performance	[199]
**Haematological Cancers**				
Acute Myeloid Leukaemia	24	WGS -RNA-seq	- Samples: tumour/normal skin - Sequencer: Illumina - Aim: Pilot, mutation landscape	[200]
Acute Myeloid Leukaemia	150	WES (50 WGS) -RNA-seq -miRNA -methylation	- Samples: tumours/normal skin - Sequencer: Illumina - Project: TCGA	[201]
Myelofibrosis	95	28-gene-panel	- Samples: patients were treated in a trial with ruxolitinib in a phase 1/2 study - Sequencer: Illumina - Aim: response to therapy, frequent mutations (*NRAS*, *KRAS*, *PTPN11*, *GATA2*, *TP53*, and *RUNX1*). Multiple mutations correlated with outcome	[202]
Myelo-Dysplasia	9	WES	- Samples: fresh specimens: low-grade disease (bone marrow mononuclear cells or peripheral-blood granulocytes); normal DNA buccal swabs/T cells - Sequencer: Illumina - Project: cancer genome project	[203]
Multiple Myeloma	133	5	- Samples: application of NGS compared to standards - Sequencer: LymphoSIGHT (Sequenta) ^2^ - Aim: prognosis/persistence of minimal residual disease	[204]
Acute Lymphoblastic Leukaemia	106	5	- Samples: application of NGS compared to standards - Sequencer: LymphoSIGHT (Sequenta) ^2^ - Trial: NCT00137111 prognosis/persistence of minimal residual disease	[205]
**Other types of cancer**				
Cutaneous Melanoma	320	WES (157 WGS) -RNA-seq -miRNA -methylation	- Samples: tumour/blood DNA - Sequencer: Illumina - Project: TCGA	[206]
Prostate Cancer	333	WES -RNA-seq -miRNA -methylation	- Samples: frozen tumours/matched blood or normal tissue - Sequencer: Illumina - Project: TCGA	[207]
Cholangio-Carcinoma	75	a- 46-gene-panel b- 236-gene-panel (introns of 19 genes)	- Samples: archival FFPE material from patients with >3 months follow-up - Sequencer: Ion Torrent and Illumina - Aim: classification/diagnostics and prognostics	[208]
Thymic Carcinoma	12	409-gene-panel	- Samples: Frozen squamous cell carcinoma/matched normal tissue (10 patients) - Sequencer: Ion Torrent - Aim: proof of concept, heterogeneous mutation landscape	[209]
Malignant Pleural Mesothelioma	123	50-gene-panel	- Samples: macro(manual)dissected specimens from FFPE samples - Sequencer: Ion Torrent - Aim: proof of concept, frequent mutation (*TP53*, DNA repair), prognosis	[210]
Adrenocortical Carcinoma	91	WES (50 WGS) -RNA-seq -miRNA -methylation	- Samples: frozen tumours/matched blood or normal tissue - Sequencer: Illumina - Project: TCGA	[211]
Merkel Cell Carcinoma	15	409-gene-panel	- Samples: polymavirus negative/CK20 negative FFPE specimens - Sequencer: Ion Torrent - Aim: driver genes exploration *TP53*, *RB1*, *BAP1*	[212]
**Paediatric oncology**				
Thyroid Carcinoma	27	50	- Samples: FFPE specimens - Sequencer: Ion Torrent - Aim: proof of concept, frequent mutations in *BRAF*, *RET* and *CTNNB1*	[213]
Thyroid Carcinomas	18	60-gene-panel	- Samples: FFPE, differentiated cancer (sporadic)/previous molecular testing - Sequencer: Ion Torrent - Aim: proof of concept, classification, prognosis	[199]
Solid Tumours (extra cranial)	100	275-gene-panel (introns of 30 genes)	- Samples: FFPE or fresh frozen (nonrhabdomyosarcoma soft-tissue, sarcoma, neuroblastoma, ewing sarcoma, osteosarcoma, rhabdomyosacroma, Wilms tumour, rare tumours) - Sequencer: Illumina - Trial (NCT01853345): actionable alterations, individualised cancer therapy	[214]
**Studies focusing on multiple Cancers**				
Various	2221	287-gene-panel (introns of 19 genes)	- Samples: FFPE samples from consecutive clinical cases of which 249 previously characterised - Sequencer: Illumina - Aim: method applicability	[215]
Colorectal, Lung Cancers	18	48-gene-panel	- Samples: archival FFPE material already genotyped - Sequencer: Illumina - Aim: NGS validation	[216]
Glioblastoma, Lung, Thyroid Cancers, Holangio - Carcinoma	986	48-gene-panel -RNA seq	- Samples: FFPE samples (both for DNA-seq and RNA-seq) - Sequencer: (anchored multiplex PCR) Illumina or Ion Torrent - Aim: proof concept and clinical applicability	[217]
Colorectal and Endometrial Cancers	32	19-gene-panel (ColoSeq)	- Samples: blood/tumour samples (MMR deficiency without hypermethylation of *MLH1* or germline MMR mutations) - Sequencer: Illumina - Aim: diagnostic/surveillance of Lynch syndrome-like (sporadic)	[218]
Over 20 Cancer types	407	50-gene-panel	- Samples: FFPE or fresh frozen (Gastric, lung, colorectal adenocarcinoma, soft tissue sarcoma, Hepatocellular carcinoma and other types) - Sequencer: Ion Torrent - Trial (NCT01853345): NEXT-1 trial. Genome-matched treatment	[219]
Various	50	WES	- Samples: 10 patients with chronic B cell lymphocytic leukaemia 20 patients with bone cancer (9 osteosarcoma, 11 chordoma). Tumour/germline DNA - Sequencer: Illumina - Project: cancer genome project	[220]
Breast, Head and Neck Cancers, Melanoma	103	236-gene-panel DNA intronic sequences from 19 genes	- Samples: reviewed own experience on FFPE material - Sequencer: Illumina - Aim: validation of clinical potential	[221]
Various solid Tumour samples	55	409-gene-panel	- Samples: FFPE from tumour (melanoma; gastrointestinal stromal, brain tumours; lung, breast, gynaecologic tract, gastrointestinal tract carcinomas) and matched normal tissues (from 20 samples). All samples previously tested for mutation landscape with a 46-gene-panel - Sequencer: Ion Torrent - Aim: validation: sensitivity, specificity, reproducibility, applicability	[222]
Various solid Tumour samples	70	46-gene-panel	- Samples: FFPE specimens previously tested genetically (*n* = 22) and 48 tested in parallel. Melanoma (*n* = 36); colorectal (16), lung (5), gastrointestinal tract (5), papillary thyroid (4), endometrial serous (3) adenocarcinomas; squamous cell carcinoma (*n* = 1) - Sequencer: Ion Torrent - Aim: validation, sensitivity, applicability, feasibility	[223]

* Other analyses: all performed using NGS; ** FFPE: formalin fixed paraffin embedded; ^1^ Adenocarc. = Adenocarcinoma; ^2^ LymphoSIGHT (Sequenta^®^) is designed to detect rearrangements (VDJ) in *IGH*, *IGK*, *TCRB*, *TCRD*, *TCRG*.

## Abbreviations

ADR	Adverse drug reaction
BRCAx	Test negative to *BRCA1*/*2* mutation
BWA	Burrows-wheeler-alignment
Bp	Base pair
ChIP-seq	Chromatin immunoprecipitation
CPIC	Clinical pharmacogenetics implementation consortium
CTC	Circulating tumour cell
ctDNA	Cell-free tumour DNA
DNA-seq	DNA sequencing
DPWG	Dutch pharmacogenetics working group
FAP	Familial adenomatous polyposis coli
FFPE	Formalin fixed paraffin embedded
Gb	Giga base
GWAS	Genome wide association studies
HBOC	Hereditary breast and ovarian cancer
ICSI	Intra cytoplasmic sperm injection
Indels	Insertion or deletion (of DNA fragments)
IVF	In vitro fertilisation
Kb	Kilo base pair
KNMP	Royal Dutch pharmacists association
MIP	Molecular inversion probe
MMR	Mismatch repair
NGS	Next-generation sequencing
PCR	Polymerase chain reaction
PGD	Preimplantation genetic diagnosis
PGS	Preimplantation genetic screening
PGH	Preimplantation genetic haplotyping
PGx	Pharmacogenetics
RNA-seq	RNA sequencing
ROI	Regions of interest
SAM	Sequence-Alignment-MAP tools
SBS	Sequencing by synthesis
SMaRT	Spliceosome-mediated RNA trans-splicing
SMRT	Single molecule real-time
SNP	Singe nucleotide polymorphism
VUS	Variant of undetermined (unknown) clinical relevance
WES	Whole exome sequencing
WGS	Whole genome sequencing

**Important definitions**	
Actionable mutation	A mutation that is potentially responsive to a targeted therapy
Coverage (in NGS)	(Or depth) the number of reads per nucleotide
Cistrome	The set of (cis-acting) DNA sequences targeted by a specific transcription factor
Depth	(Or coverage) the number of reads per nucleotide
Diagnostic yield	The likelihood that a given test will return information that helps establishing a diagnosis
Driver mutation	A somatic mutation OCCURRING in cancer that is implicated in oncogenesis
Driver gene	A gene where driver mutations are frequently found
Effect-size (of a gene variant)	The measurement of the magnitude of the effect of a gene variant
FastQC	Application handling raw-sequence data from high throughput NGS sequencers
Genetic risk modifier	A gene variant with no intrinsic pathogenic action and a small effect-size, but that can modify the penetrance of a co-existing pathogenic mutation
Passenger mutation	A somatic mutation (found in cancer but not only) that has no implication in oncogenesis and does not give growth advantage
Penetrance (genetics)	The proportion of INDIVIDUALS carrying a gene variant (or mutation) and expressing the phenotype associated to the variant
Sequencing by synthesis (in NGS)	method developed by Solexa^®^ and currently used by Illumina^®^ in which each dNTP with a fluorescent reversible terminator is added separately, then is cleaved to allow the incorporation of the next base
Single molecule real-time (in NGS)	In this method, several DNA polymerase enzymes are contained in separate nanophotonic structures where a single stranded DNA is also incorporated. DNA synthesis is made using differentially labeled nucleotides
Specificity (in NGS)	The amount of regions of interest (ROI) theoretically captured for a NGS analyses that are correctly enriched and sequenced
Spliceogenic variant	A gene variant causing a splice alteration
Targeted re-sequencing	Isolation and sequencing of a subset of genes or regions of interest. Different approaches can be used (see the text)
Theranostic	This is a sort of diagnostic test that can be used to select a targeted therapy
Translatome	All mRNA fragments that are translated in a moment or in a condition.
Variant of undetermined/unknown clinical significance (VUS)	A rare genetic variant for which pathogenicity was neither confirmed nor excluded
